# Automated Bi‐Ventricular Segmentation and Regional Cardiac Wall Motion Analysis for Rat Models of Pulmonary Hypertension

**DOI:** 10.1002/pul2.70092

**Published:** 2025-05-12

**Authors:** Marili Niglas, Nicoleta Baxan, Ali Ashek, Lin Zhao, Jinming Duan, Declan O'Regan, Timothy J. W. Dawes, Chen Nien‐Chen, Chongyang Xie, Wenjia Bai, Lan Zhao

**Affiliations:** ^1^ National Heart and Lung Institute Imperial College London London UK; ^2^ Biological Imaging Centre Imperial College London London UK; ^3^ School of Computer Science University of Birmingham Birmingham UK; ^4^ MRC London Institute of Medical Sciences Imperial College London London UK; ^5^ Department of Computing Imperial College London London UK; ^6^ Department of Brain Sciences Imperial College London London UK; ^7^ Data Science Institute Imperial College London London UK

**Keywords:** 3D motion analysis, cardiovascular disease, deep learning, magnetic resonance imaging, pulmonary hypertension

## Abstract

Artificial intelligence‐based cardiac motion mapping offers predictive insights into pulmonary hypertension (PH) disease progression and its impact on the heart. We proposed an automated deep learning pipeline for bi‐ventricular segmentation and 3D wall motion analysis in PH rodent models for bridging the clinical developments. A data set of 163 short‐axis cine cardiac magnetic resonance scans were collected longitudinally from monocrotaline (MCT) and Sugen‐hypoxia (SuHx) PH rats and used for training a fully convolutional network for automated segmentation. The model produced an accurate annotation in < 1 s for each scan (Dice metric > 0.92). High‐resolution atlas fitting was performed to produce 3D cardiac mesh models and calculate the regional wall motion between end‐diastole and end‐systole. Prominent right ventricular hypokinesia was observed in PH rats (−37.7% ± 12.2 MCT; −38.6% ± 6.9 SuHx) compared to healthy controls, attributed primarily to the loss in basal longitudinal and apical radial motion. This automated bi‐ventricular rat‐specific pipeline provided an efficient and novel translational tool for rodent studies in alignment with clinical cardiac imaging AI developments.

**Abbreviations**
2D: two‐dimensional
3D: three‐dimensional
AI: artificial intelligence
BSA: body surface area
CO: cardiac output
ECG: electrocardiogram
ED: end‐diastolic
EDV: end‐diastolic volume
EF: ejection fraction
ES: end‐systolic
ESV: end‐systolic volume
FCN: fully convolutional network
HR: high resolution
LR: low resolution
LV: left ventricular
M: mass
MCT: monocrotaline
MR: magnetic resonance
PAH: pulmonary arterial hypertension
PH: pulmonary hypertension
RV: right ventricular
SuHx: Sugen hypoxia
SV: stroke volume

## 1 Introduction

Cardiac magnetic resonance (cardiac MR) imaging has become the gold standard technique to assess cardiac function, allowing noninvasive and detailed structural, functional, haemodynamic, and molecular characterization of both ventricles in a cardiovascular disease setting. However, cardiac MR image processing is time consuming and requires expertise for accurate endo‐ and epicardial delineation. Artificial intelligence (AI) techniques, in particular deep learning methods, have been developed for human and patient cohort cardiac MR image analysis to provide efficiency and consistency to the task for clinical studies [1].

AI methods have shown their value in phenotyping ventricular changes/remodeling and predicting survival outcomes of patients with pulmonary arterial hypertension (PAH). These methods provide accurate image analysis of the right ventricle and allow for three‐dimensional (3D) mapping of cardiac motion, emphasizing the heterogeneous pattern of ventricular contraction [2, 3]. PAH may have poor outcomes, and the adaptation of the right ventricle is an independent predictor of survival for PAH patients [4]. 3D cardiac models offer a more sensitive prognostic marker than other traditional cardiac MR‐derived metrics, such as end‐diastolic volume and ejection fraction [2, 3] and allow a comprehensive understanding of how the complex right ventricular (RV) motion traits contribute to cardiac performance. Furthermore, 3D cardiac motion models have shown their ability to identify physiological features portending poor outcomes before the manifestation of overt heart dysfunction and require lower patient numbers for genotype‐phenotype associations [5].

Rodent models are crucial for gaining insight into morphological and pathophysiological changes occurring in cardiovascular diseases, contributing to biomarker and therapy discovery. Cardiac MR has been applied in preclinical models, initially for the serial assessment of left ventricular (LV) changes, and more recently to follow RV remodeling throughout disease progression examinations [6, 7, 8, 9]. Rodent‐specific algorithms have been introduced to automate the cardiac MR labeling process. Currently, a limited number of algorithms exist that target rodent models, specifically focusing on either left endocardial border segmentations [10, 11] or bi‐ventricular segmentations in mouse models [12]. To date, no efficient pipelines that include RV annotations have been developed for rats, even though rat experimental models are preferred in the pulmonary hypertension (PH) research field, with 93% of preclinical PH papers reporting studies conducted on rats [13].

The primary aim of this study was to establish a deep learning pipeline that generates fast and accurate bi‐ventricular segmentations of rat cardiac MR images. The performance of the rat‐specific model was tested on both male and female healthy and PH rat models. Furthermore, we aimed to implement the regional cardiac motion phenotyping established in the clinical setting for patients into our rat PH models by fitting a rat heart atlas to the automated segmentations to map 3D cardiac motion patterns. To capture bi‐ventricular remodeling, especially the impact of disease progression on the right ventricle, we utilized two of the most established PH rat models, monocrotaline (MCT) and Sugen hypoxia (SuHx), to train and evaluate the pipeline. The pipeline provided an efficient and unique tool for rodent cardiac MR image analysis, with the potential to bridge the understanding of PH morphological traits made in human populations with rodent models.

## 2 Methods

### 2.1 Animal Models

All animal experiments were approved by the Imperial College London Ethical Committee and conducted in accordance with the UK Home Office Animals (Scientific Procedures) Act 1986 (London, United Kingdom). Adult Sprague Dawley or Wistar Kyoto rats (Charles River, UK) were used. The rats were housed in 12‐h light/dark cycles with ad libitum access to water and food under standard laboratory conditions.

To develop a bi‐ventricular segmentation network, we utilized two PH rat models to prompt ventricular remodeling: (i) subcutaneous injection of monocrotaline (60 mg/kg; Sigma‐Aldrich), and (ii) subcutaneous injection of Sugen (SU‐5416; 20 mg/kg; Sigma‐Aldrich) followed by 4 weeks of hypoxia exposure (F_i_O_2_ = 10%) and then return to room air for 4 weeks. Animals weighed approximately 180–220 g at the start of the experiment and were approximately 6 weeks of age. Longitudinal cardiac MR examinations were performed at baseline (0 weeks) and at 2‐ and 4‐weeks post MCT injection and at 4‐, 6‐ and 8‐weeks post SU5416 injection (Supporting Information S1: Figure S1, in supporting material).

### 2.2 Cardiac Magnetic Resonance Examination

#### 2.2.1 Image Acquisition

To gain morphological and functional information on ventricular remodeling, serial cardiac MR scans were obtained. Cardiac MR images were acquired in the Biological Imaging Centre at Imperial College London on a 9.4 T Bruker scanner (Bruker BioSpec, Ettlingen, Germany). The MR scanner was equipped with a volume transmit coil combined with a rat cardiac array receiver. The data was acquired with Paravision 7.0. Anesthesia was induced by 3%–5% isoflurane and maintained at 1.5%–2% for the duration of the cardiac MR scan. To maintain physiological conditions throughout, anesthesia was adjusted to maintain a respiratory rate between 40 and 60 breaths per minute. A three‐lead electrocardiogram (ECG) was positioned subcutaneously near the right forelimb, the left forelimb and right hindlimb for continuous heart rate monitoring throughout the cardiac MR examination. The rats were placed on a dedicated rat bed onto a pressure sensor for respiratory motion detection. The body temperature was monitored by a rectal temperature probe (SA Instruments, Stony Brook, NY, USA) and maintained at approximately 36°C–37°C using a heating mat.

Cardiac MR acquisition protocols were customized to align with the standardized protocols for human cardiac MR [14] as described in [15]. Prospective R‐wave‐based cardiac and respiratory triggering was used to acquire the cardiac MR images. A T_1_‐weighted gradient echo fast low angle shot (FLASH) sequence was used to acquire 2D multi‐slice stack cinematographic (cine) images. Parameters were set as follows: repetition time (TR) = RR interval/number of frames ( ~6.2 ms for ~27 frames); TR_effective_ = RR interval; echo time (TE) = 2.3 ms; flip angle = 18°, in‐plane spatial resolution = (200 × 200) μm^2^; slice thickness = < 1.5 mm; scan time = < 20 min. Acquisition matrix size was 192 by 192. Slice thickness was adjusted to cover both ventricles, and the slices were placed to cover the following anatomical landmarks: the apex and base of the ventricles, the aortic valve and the top of the LV wall.

3D cardiac MR images were acquired to gain a more detailed morphological definition in the ventricular long axis direction. The images were acquired using a 3D FLASH sequence with TR = 7.3 ms, TE = 2.4 ms, flip angle = 28°, total scan time = < 40 min per phase, and resolution = (200 × 200 × 340) μm^3^. Only the end‐diastolic (ED) and end‐systolic (ES) phases of the cardiac cycle were imaged.

The cine images (also referred to as low‐resolution; LR) were acquired as part of all routine cardiac examinations. The images have a relatively large slice thickness (< 1.5 mm), which may lead to a staircase effect in the long‐axis direction. Areas particularly vulnerable to information loss are the apex and the base of the right ventricle as they have a higher/steeper curvature. The 3D cardiac MR images (also referred to as high‐resolution; HR) have a fourfold higher resolution in the long‐axis direction (340 µm).

#### 2.2.2 Cardiac MR Image Manual Segmentation

Deriving valuable indices from cardiac MR images requires manual segmentation of the gray‐scale images at ED and ES phases of the cardiac cycle. The ED frame was defined as the first frame of each cine image sequence [16]. The ES frame was determined, first, by the closure of the mitral valve and, second, by the smallest LV cavity (blood pool) size at the mid‐ventricular level. The RV epi‐ and endocardial border extended from the ventricular aspect of the tricuspid and pulmonary valves to the apex, and the LV borders extended from the mitral and aortic valves to the apex [17, 18]. The interventricular septum was considered part of the LV wall. Trabeculae and papillary muscles were included in the blood pool for simplicity and applicability. Images were segmented by an observer with 4 years of experience in cardiac segemntation. The manual segmentations were performed using the free publicly available software ITK‐SNAP [17] (www.itksnap.org).

### 2.3 Pipeline

**Figure 1:**
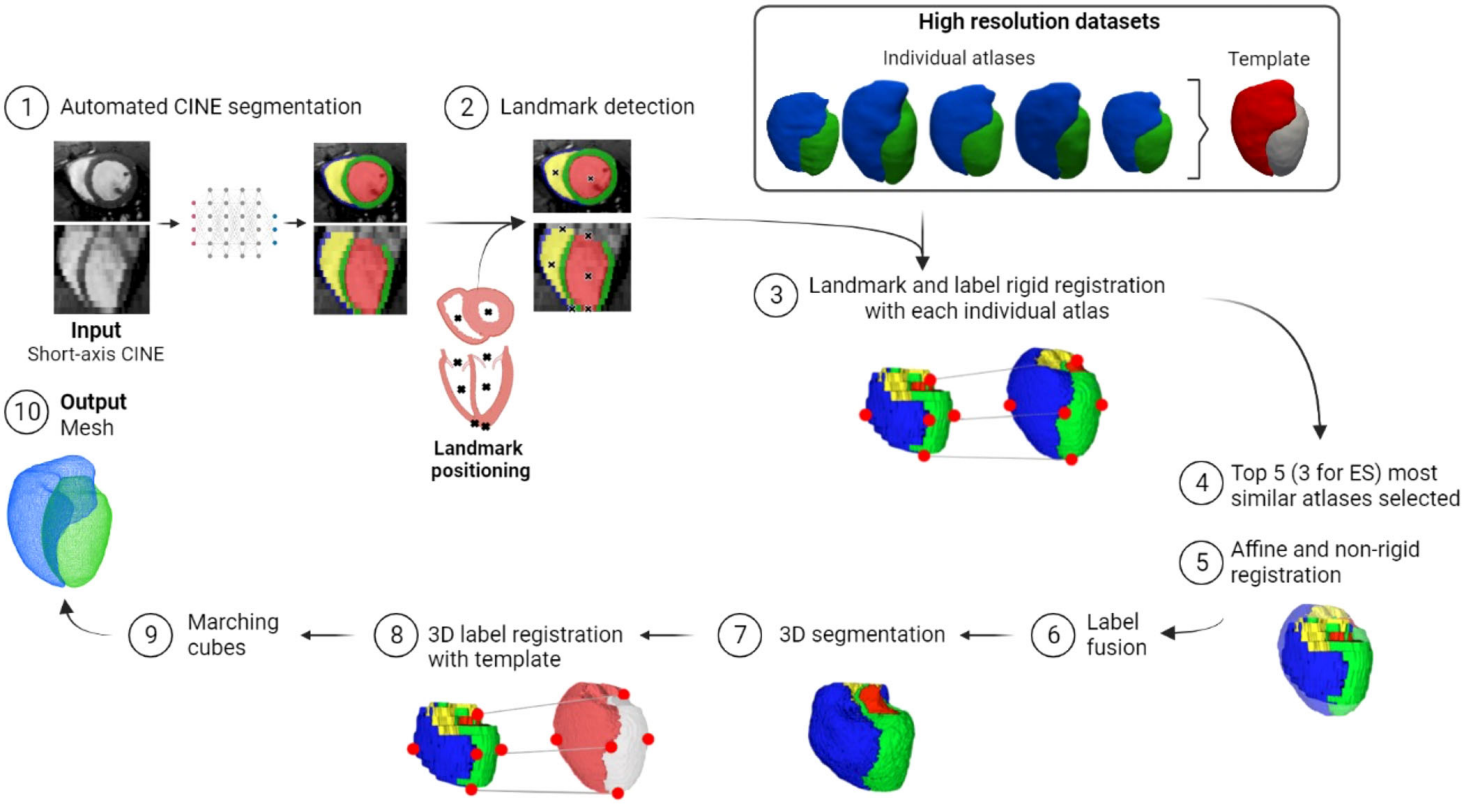
Automated cine segmentation and 3D mesh generation pipeline. The trained fully convolutional network predicts the initial segmentation onto the cine short‐axis stack end‐diastolic (ED) and end‐systolic (ES) phase input images, followed by landmark detection and multiple registration steps. Multiple most similar atlases were averaged in label fusion and subsequently re‐registered to an average template label to allow inter‐subject comparison. Finally, a marching cubes algorithm was applied to generate right ventricular (RV) and left ventricular (LV) meshes.

We adapted a pipeline from human cardiac MR application [19] for segmentation and generation of 3D cardiac mesh models using cardiac MR images from both control healthy and PH rat models. In the first stage, a trained fully convolutional network (FCN) predicted an initial segmentation on short‐axis stack cine images. In the second stage, the predicted segmentations were each co‐registered with the most similar HR atlas to generate anatomically accurate HR segmentations and subsequent mesh models. The workflow of the full pipeline is described in Figure 1.

#### 2.3.1 Input Data for Fully Convolutional Model Development

A total of 163 short‐axis stack cine scans were acquired from healthy and ventricular remodeling‐induced animal models. These scans included data from healthy rats (*n* = 27) and pathological groups of MCT 2‐weeks (*n* = 26), MCT 4‐weeks (*n* = 26), SuHx 4‐weeks (*n* = 12), SuHx 6‐weeks (*n* = 50) and SuHx 8‐weeks (*n* = 22) (Table 1). The images and corresponding manual segmentations were randomly split into 98 training, 18 validation, and 47 testing sets (60/10/30 split ratio) in a stratified manner. The proportions of the number of scans for each timepoint were maintained in the groups. The validation set included a data set randomly chosen from each timepoint (Table 1). Splitting was conducted subject‐wise, all images from one rat were used only once in either the training, testing or validation group. Only images from the ED and ES phases of the cardiac cycle were used. The deep learning model was trained on the training and validation sets, and the performance of the model was evaluated on the test set. All results reported on the deep model's performance and subsequent further analysis is derived from the test set.

**Table 1:** Number of subjects used in training, validation, and test cohorts. The subject datasets were comprised of cine end‐diastolic (ED) and end‐systolic (ES) cardiac MR images along with the corresponding manual segmentations. Data was acquired from monocrotaline (MCT) and Sugen hypoxia (SuHx) rat models at different time points.

	0 w	MCT	MCT	SuHx	SuHx	SuHx	Total
		2 w	4 w	4 w	6 w	8 w	
Train	16	16	16	7	30	13	98
Validation	3	3	3	1	5	3	18
Test	8	7	7	4	15	6	47
Total	27	26	26	12	50	22	163

#### 2.3.2 Preprocessing

All images were pre‐processed before training and deploying the FCN models to ensure that they fit the network architecture and that the intensities were comparable between subjects. Image intensity was adjusted by discarding the 1% and 99% of intensity values and rescaling the remaining intensities between values 0 and 1.

Data augmentation enables to increase the variety of data the network can learn from modifying copies of existing images by making minor changes. On‐the‐fly data augmentation was performed during training, consisting of random intensity rescaling (±10%), rotations (±10°), and spatial shift translations along one image axis (*x*, *y*, or *z* axis; ±10 pixels) (Supporting Information S1: Table S1, in supplementary). The augmentation were applied in an automated manner with independent transformation parameters for each image, on a per‐training‐batch basis (training batch size was two datasets). Image loading, pre‐processing and data augmentation functions were implemented using Python with the libraries numpy, nibabel, scipy and SimpleITK.

#### 2.3.3 Overview of the Deep Learning Network Architecture

In the first stage of the pipeline, we adapted a fully convolutional network (FCN) previously developed for human cardiac MR image segmentation with an encoder‐decoder structure [18]. We utilized a 2D network architecture rather than the 2.5D network used in [18], as previous experiments indicated that with our rat datasets with a smaller number of training datasets, the 2D architecture outperformed the 2.5D network (Supporting Information S1: Figure S2, in supplementary). The network encoder path was comprised of 14 3 × 3 convolutional layers, each with a batch normalization and a rectified linear unit activation function. Feature maps were extracted from fine to coarse levels with five strided convolutional downsampling layers. The decoder path used transposed convolutional layers to upsample the feature maps to the original image resolution size (Supporting Information S1: Figure S3, in supplementary). The cross‐entropy loss function is defined, comparing the predicted probability map to the manual segmentation. The loss function was minimized by the Adam stochastic gradient descent algorithm.

The FCN was trained for 200 epochs with a batch size of 2. The learning rate was set to 0.001. The model was implemented using TensorFlow. Model training took ~1.5 h on an NVIDIA GeForce RTX 2080 Ti GPU.

#### 2.3.4 Atlas Fitting

A HR atlas refinement step was utilized with anatomical shape prior knowledge via atlases–manually labeled 3D cardiac MR image segmentations (Table 3). A multi‐atlas label fusion method was used to predict the 3D label on the estimated cine segmentation from the FCN.

A cine stack manual segmentation label was first rigidly registered with all 3D manual labels, which was initiated by the six bi‐ventricular landmarks. The landmarks were automatically placed in the center of the apical, basal, and mid‐ventricular level slices on bi‐ventricular segmentations of the predicted as well as the HR atlas labels. This ensured that the atlases were aligned in the same spatial coordinate system. Out of a pool of 17 ED and 8 ES HR datasets (Table 3), the most similar atlases (five for ED and three for ES phase) were taken for each subject based on label consistency scores. An affine and free‐form deformation based nonrigid registration was used to warp the selected atlases to the subject locally, which were then fused to make one final 3D segmentation. To ensure inter‐subject comparison of wall motion results, all 3D labels from each subject were co‐registered, using rigid registration, to an average template atlas created from the HR datasets. This average ventricular template image was generated using all ED and ES 3D datasets.

To model ventricular surface topologies, the marching cubes algorithm was applied to the predicted 3D label to produce a regular epi‐ and endocardial triangular surface mesh. The resultant mesh of 18,312 and 14,440 points was produced for the RV and LV epi‐ and endocardial borders at the ED and ES phases of the cardiac cycle, respectively.

### 2.4 Automated Segmentation Evaluation

To assess the performance of the pipeline, the automated segmentations were compared to manual segmentations using the segmentation label accuracy and cardiac MR segmentation derived indices. The accuracy of the automated segmentation labels was assessed using the Dice similarity coefficient. This measures the overlap between the ground‐truth (manual) segmentation and the predicted (automated) segmentation and is calculated as follows:

$$\text{Dice}=\frac{2|S_{1}\cap S_{2}|}{\left|S_{1}\right|+|S_{2}|},$$

where S1 and S2 denote the segmentations from two methods. The Dice metric ranges from 0 to 1, where 1 indicates perfect overlap.

Additionally, segmentation accuracy was evaluated using cardiac MR‐derived indices. Stroke volume (SV) was defined as the difference between ED volume (EDV) and ES volume (ESV); SV = EDV – ESV. Cardiac output (CO) was derived from the following calculation: CO = heart rate * SV/1000, units are L/min. The EF was defined as EF = SV/EDV * 100%. Myocardial mass was calculated by multiplying the specific myocardial density (1.05 g/mL) by the ED myocardial volume. All parameters were indexed to the body surface area (BSA). This was calculated according to Meeh's formula, BSA = k * W2/3/10000, where k is a constant 10, W is body weight in grams and the resulting BSA is in m^2^. The difference in cardiac indices between manual segmentation and automated segmentation was calculated.

In addition to testing the model's performance on a held‐out test cine data set that included male control, MCT and SuHx rats, the segmentation model was further assessed on a data set containing female rat control, MCT and SuHx groups. The objective was to assess the generalizability of the datasets on an unseen pathological model.

#### 2.4.1 Interobserver Variability

To assess interobserver variability, 20 scans were used. Inter‐operator variability was assessed between an operator with 4 years of experience and another operator with 10 years of experience in cardiac segemntation. The manual segmentation between both operators was evaluated using the Dice correlation coefficient metric.

### 2.5 Regional Wall Motion Analysis

Regional wall motion from endocardial meshes was calculated for each mesh point as the distance between the ED and ES phases from the endocardial meshes for both ventricles. The magnitude of motion was based on a root mean square of the three motion components–longitudinal, radial, and circumferential motions (Supporting Information S1: Figure S4, in supplementary). Longitudinal motion was calculated as the difference between a corresponding point in the z‐axis plane (basal to apical). Radial motion was calculated as the distance traveled by a point between the ED and ES in relation to the centreline. The centreline was defined as a line along the septal wall in the longitudinal direction of the ventricles. By assuming a circular cross‐sectional shape, the angular change between the ED and ES points was calculated as a distance and presented as the circumferential motion.

Regional motion was assessed from 28‐segments across the left and right ventricle using two‐way ANOVA. To accomplish this, a custom partitioning of the LV and RV regions was defined. The left ventricle was partitioned into 17 segments, and the right ventricle was partitioned into 11 segments from the 3D cardiac mesh (Supporting Information S1: Figure S5A, in supporting material). Apart from the apex, which was the tip of the ventricle, the basal, mid, and apical areas were considered thirds along the longitudinal direction. Further partitioning occurred from the insertion points every 60°, defining regions into anterior, anterolateral, inferolateral, inferior, inferoseptal, and anteroseptal regions (with the last two regions applicable only for the left ventricle. The selected regions from the 3D mesh were also represented as a bullseye plot (Supporting Information S1: Figure S5B,C, in supplementary). For regional assessment, the median motion for each section per subject was averaged in correspondence to cardiac MR scan timepoints.

### 2.6 Statistical Analysis

The results are reported as the mean ± standard deviation unless the results had a non‐normal distribution, for which the median with interquartile range was reported. The FCN algorithm results were compared to manual segmentations using Dice correlation coefficients and cardiac parameters via two‐way ANOVA and Bland‒Altman plot analysis in GraphPad Prism 8. Comparisons between the different timepoints were calculated as percentage differences from the 0‐week group.

## 3 Results

### 3.1 Fully Convolutional Network Development and Performance

#### 3.1.1 Automated Segmentation Performance

The fully convolutional network (FCN) established in this study was based on a data set of 163 short‐axis cine cardiac MR scans collected longitudinally from MCT and SuHx PH rats (Table 1). The model produced a segmentation in < 1 s (mean 0.10 ± 0.20) for each cardiac phase at end‐diastole (ED) and end‐systole (ES). In comparison, the manual segmentation of the left and right ventricle of one subject at ED and ES phases is user‐dependent and may require as much as 20 min.

#### 3.1.2 Automated Segmentation Accuracy

**Figure 2:**
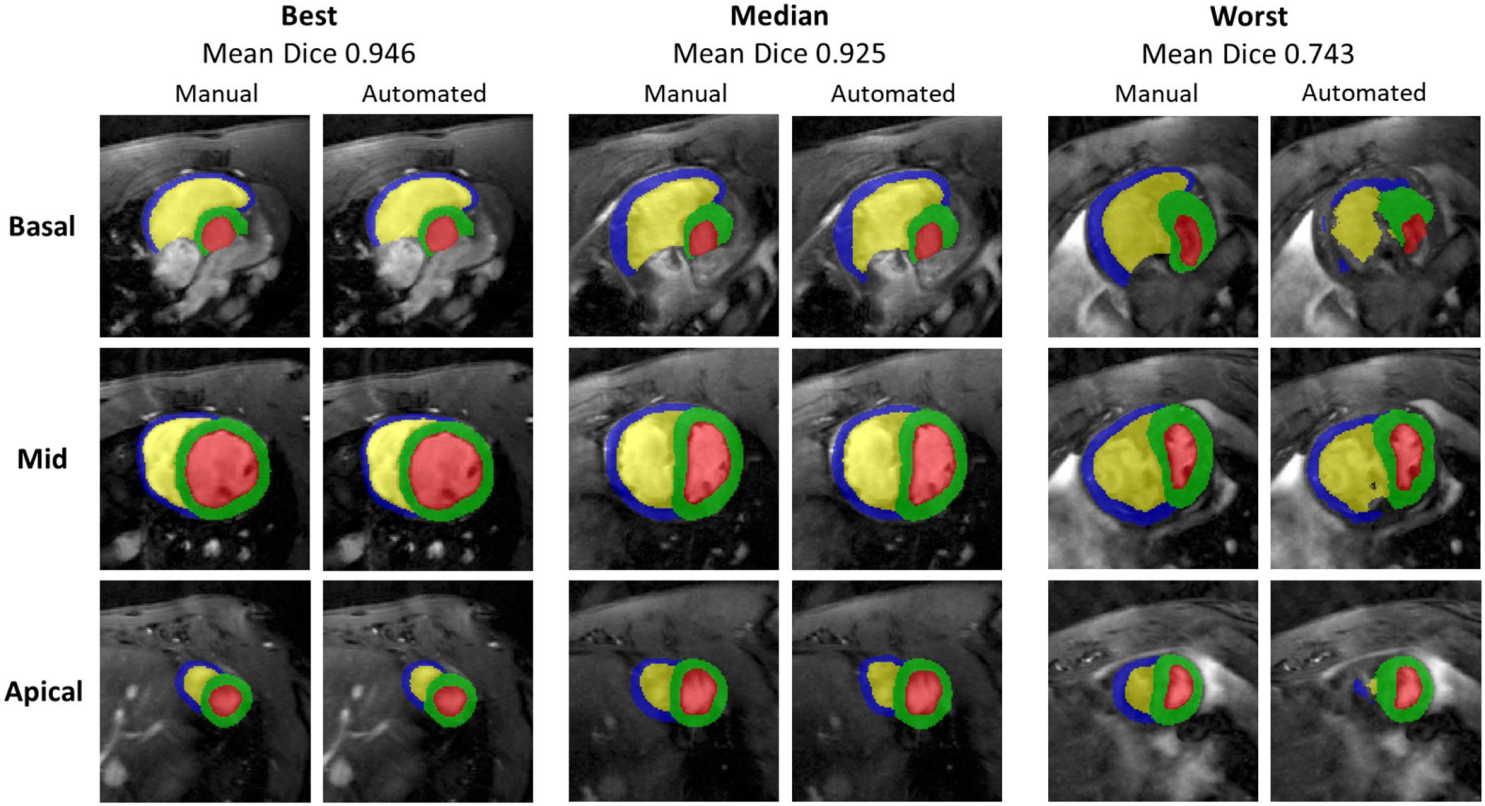
Visual comparison of the best, median and worst mean Dice correlation coefficient cases from manual and automated end‐diastolic segmentations. Mean dice was calculated as the average of all four labels. Each case is displayed by the basal, mid‐ventricular and apical slices (from top to bottom) to indicate differences.

Good agreement was observed across all four segmentation labels (Figure 2). Three labels demonstrated a mean Dice value above 0.92 (Table 2) whereas the RV myocardial label achieved a mean value of 0.836. The apical and basal slices showed the most variability. The ED segmentation showed higher agreement between the manual and automated segmentation methods compared to the ES segmentation, as indicated by the Dice metric (Table 2). Slice‐by‐slice comparison of the model's performance on RV blood pool and RV wall indicated that the most differences between the automated and manual segmentations were in the basal and apical slices (Supporting Information S1: Figure S6, in supplementary), as seen visually from Figure 2.

**Table 2:** Dice correlation coefficient for each end‐diastolic (ED) and end‐systolic (ES) label. The mean and standard deviation (SD) were calculated across all datasets for end‐diastolic and end‐systolic right (RV) and left ventricular (LV) labels.

Phase	Label	Dice	
		Mean	SD
Diastole	LV cavity	0.967	0.029
	LV wall	0.917	0.034
	RV cavity	0.937	0.048
	RV wall	0.821	0.072
Systole	LV cavity	0.925	0.044
	LV wall	0.901	0.035
	RV cavity	0.919	0.037
	RV wall	0.821	0.063

Of the 47 datasets tested to validate the automated segmentation method, only three failed due to inaccurate predictions in the basal and apical slices. Main factors leading to this segmentation failure where attributed to i) low contrast in the blood pool cavity caused by turbulent blood flow and ii) brighter background due to the presence of edema surrounding the heart (Supporting Information S1: Figure S7, in supplementary). These three datasets were excluded from the analysis, and all subsequent analysis was conducted using the test data set with a sample size of 44.

#### 3.1.3 Physiological Cardiac Parameters

**Figure 3:**
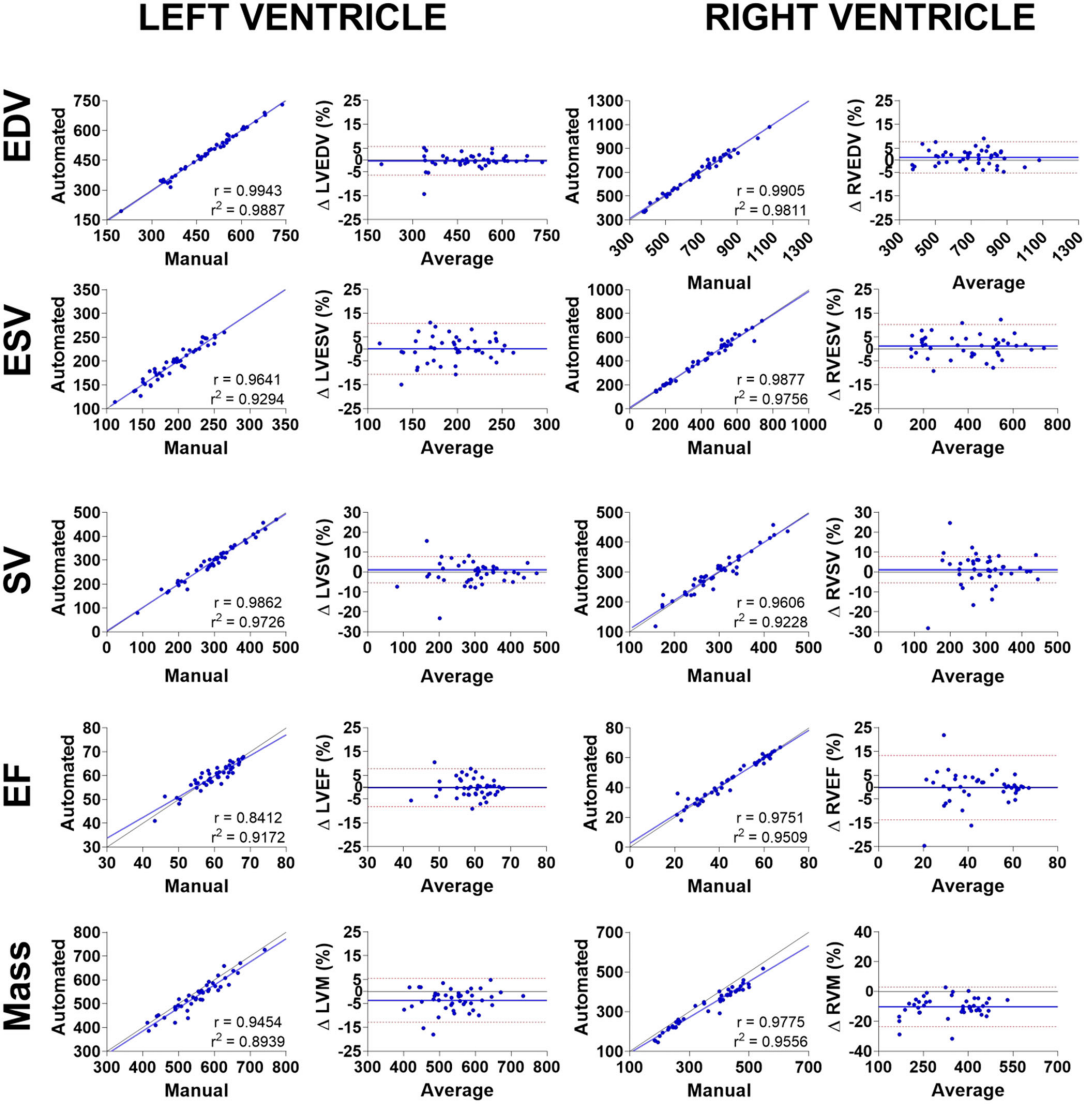
Comparison of the right and left ventricular traditional cardiac metrics as calculated from the manual and automated segmentation methods. End‐diastolic volume (EDV; first row), end‐systolic volume (ESV; second row), stroke volume (SV; third row), ejection fraction (EF; fourth row) and myocardial mass (fifth row) values were assessed from the left and the right ventricles. Respective correlation plots (first and third columns) and Bland‐Altman plots (second and fourth columns) are shown. Bland‐Altman plots include the line of equality as a continuous black line, average bias between the methods as a continuous blue line, limits of agreement as interrupted red lines. Sample size for all analysis was *n* = 44.

Additionally, the agreement between manual and automated methods was further evaluated using volume‐based measures extracted from MR‐derived cardiac indices such as EDV, end‐systolic volume (ESV), stroke volume (SV), ejection fraction (EF) and mass (M). Overall, the correlation coefficient (*r*) for all volumetric and functional values was high, with the lowest value observed for LVEF (*r* = 0.9106), indicating a strong association between manual and automated values (Figure 3). Additional Bland‒Altman analysis further confirmed the consistency between the methods, showing minimal bias and narrow limits of agreement across most indices (Figure 3). The EDV, ESV, SV and EF values displayed good agreement for both ventricles, as the bias between methods was less than 1.5% on average (Supporting Information S1: Table S2, in supplementary). However, our automated segmentation method underestimated both myocardial masses–10.3% ± 6.7% for RVM (*p* < 0.0001) and 3.7% ± 4.7% for LVM (*p* < 0.0001).

#### 3.1.4 Interoperator Variability Analysis

The interoperator variability was assessed to provide a reference for comparing the automated results. Overall, the automated method outperformed the human readers in all compared cardiac indices except ventricular mass (Supporting Information S1: Figure S8, in supplementary). Specifically, lower bias and variability in EDV, ESV and EF values were observed compared to human readers (5.7 ± 23.9 µL vs. 34.2 ± 28.6 µL, −0.6 ± 28.3 µL vs. 37.1 ± 26.9 µL, 0.8 ± 3.7% vs. −2.6% ± 3.8%, respectively). The RV myocardial wall segmentation was more accurate between the segmentations from the two readers. R1's comparison with the automated values showed lower bias and smaller standard deviations in contrast to R2 when evaluated against automated values; yet the magnitude of bias observed between R1 and R2 was similar to that of R2 and the automated bias values (Supporting Information S1: Table S3).

#### 3.1.5 Model Performance Across Various Degrees of RV Remodeling

**Figure 4:**
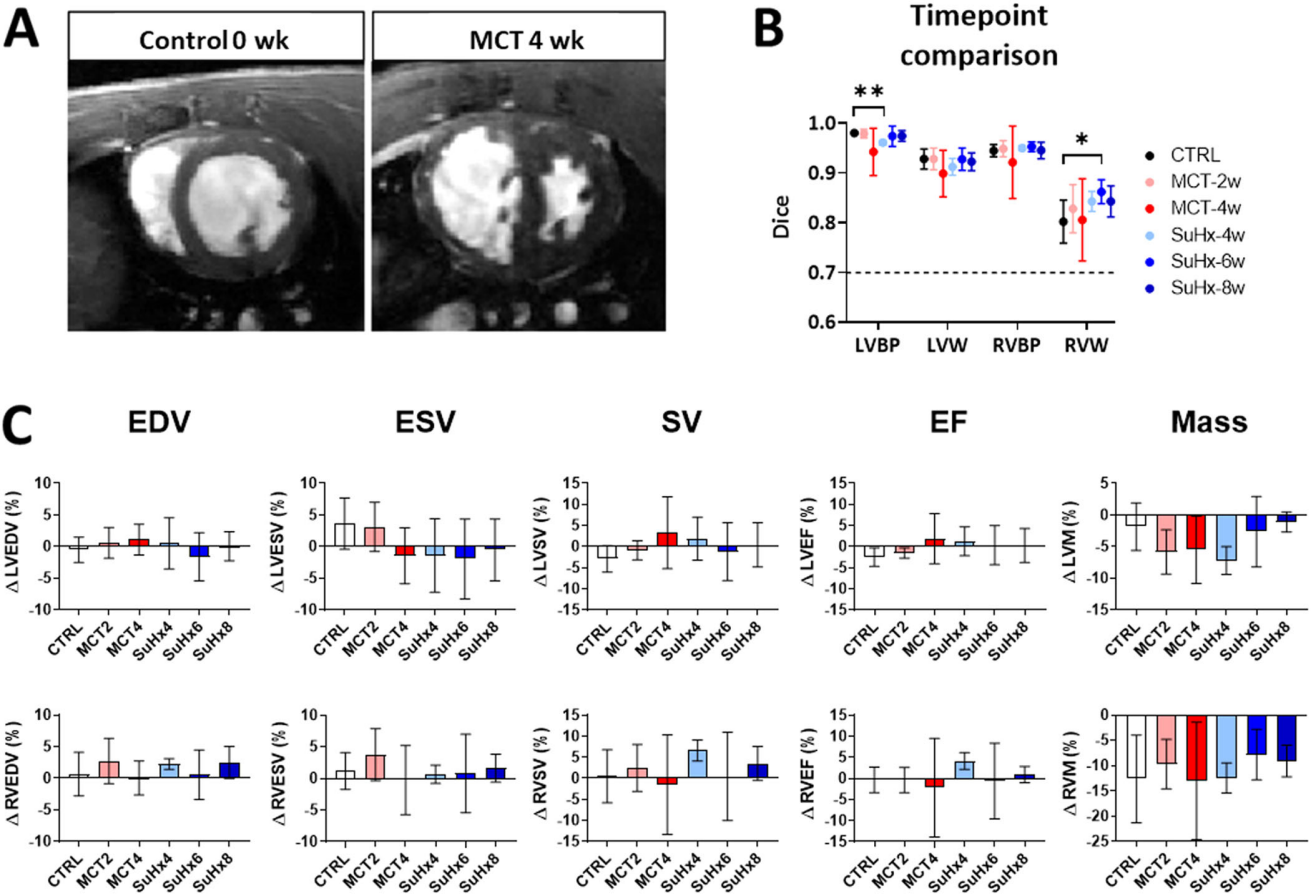
Comparison of our model's performance across healthy and diseased rat groups. (A) Representative cardiac magnetic resonance images from mid‐ventricular level of healthy (0 week) and diseased timepoint with severe ventricular remodeling (monocrotaline (MCT)‐induced 4‐week timepoint). (B) Groupwise comparison of segmentation accuracy using Dice metric for all four labels of left ventricular (LV) and right ventricular (RV) blood pool (BP) and myocardial wall (W). Healthy, monocrotaline (MCT) and Sugen hypoxia (SuHx) acquired datasets were used. We observed that the SuHx LV blood pool (at 4‐weeks) and RV wall (at 6‐weeks) displayed differences in accuracy when compared to control. (C) Percentage changes between manual and automated segmentation derived end‐diastolic volume (EDV; first column), end‐systolic volume (ESV; second column), stroke volume (SV; third column), ejection fraction (EF; fourth column) and myocardial mass (fifth column) values were assessed from the left (top row) and the right ventricles (bottom row). Sample size for all analysis was *n* = 44. Significance levels were defined as *p < 0.05; **p < 0.01.

MCT and SuHx models displayed ventricular structural remodeling typical of PH development, primarily characterized by hypertrophy of the RV wall and trabeculae, RV dilatation, and septal wall flattening (Figure 4A). As such, group‐wise comparisons between healthy and PH‐rat cohorts were performed to assess the model's performance throughout the spectrum of ventricular remodeling. The cohort comparison revealed that all timepoint groups were segmented at a similar standard, with good agreement across all investigated cardiac indices (Figure 4B,C). The percentage differences obtained through manual and automated methods were small and consistent across all timepoints. However, it is noteworthy that the EF measurement at MCT 4‐week and SuHx 6‐week timepoints had higher variability.

#### 3.1.6 Female Rat Data Set

To evaluate generalizability, the FCN performance was assessed using a separate data set that included female controls, MCT and SuHx, which had not been used in the model training. Our results indicated that, similar to male datasets, the segmentation accuracy was high for all labels (Supporting Information S1: Figure S9A, supplementary) with lowest values observed only from the RV myocardial wall labels (Dice 0.85 ± 0.047 for ED and 0.82 ± 0.083 for ES phases). All volumetric and functional measurements were predicted with good agreement. Nonetheless, the myocardial mass values for both the left and right ventricle were systematically underestimated by the model (−17.7 ± 10.42 mg for LVM and −26.4 ± 11.65 mg for RVM; *p* < 0.0001) (Supporting Information S1: Figure S9B, supplementary).

### 3.2 3D Cardiac Modeling

A pool of 17 ED and 8 ES high resolution segmentations (Table 3) were used as atlases to map the predicted deep learning segmentation to produce 3D cardiac motion meshes and corresponding wall motion maps.

**Table 3:** Number of subjects acquired and compiled into a 3D high‐resolution atlas. The subject datasets were comprised of end‐diastolic (ED) and end‐systolic (ES) 3D cardiac MR images along with the corresponding manual segmentations. Data was acquired from monocrotaline (MCT) and Sugen hypoxia (SuHx) rat models at different time points.

	ED	ES
Control	4	1
MCT 2 w	3	
MCT 4 w	4	1
SuHx 4 w	2	2
SuHx 6 w	2	2
SuHx 8 w	2	2
Total	17	8

#### 3.2.1 Regional Wall Motion

**Figure 5:**
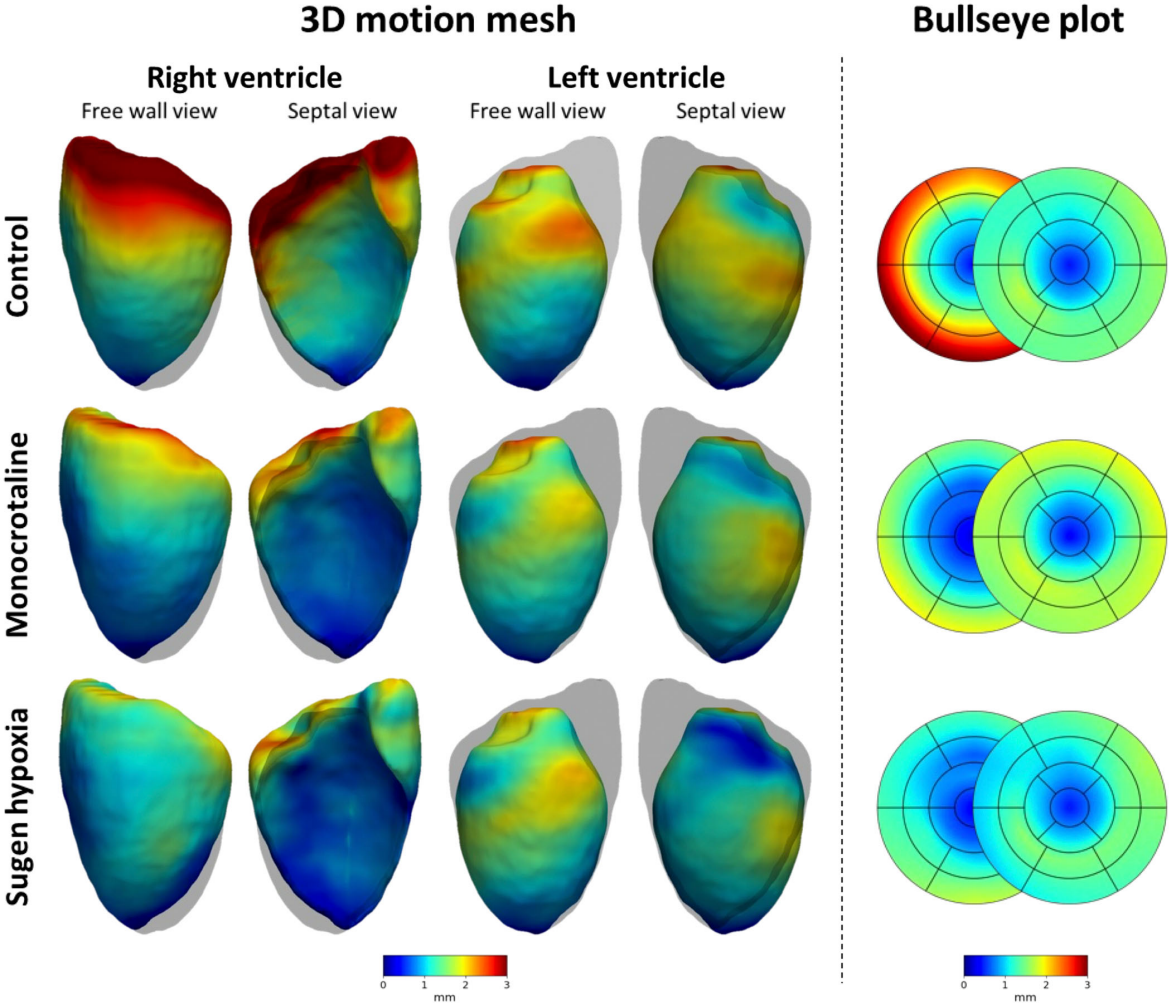
Regional cardiac motion derived from endocardial wall movement between end‐diastole and end‐systole in rat models. The average right (RV) and left (LV) ventricular regional wall motion patterns displayed on a template 3D mesh. The magnitude of wall motion (mm) is shown in control, monocrotaline (MCT, 4‐week) and Sugen hypoxia (SuHx, 4‐week) rat models. The corresponding regional wall motions for control, MCT and SuHx animals are also displayed on the 28‐segment model (17‐segments for the left and 11‐segments for the right ventricle).

**Figure 6:**
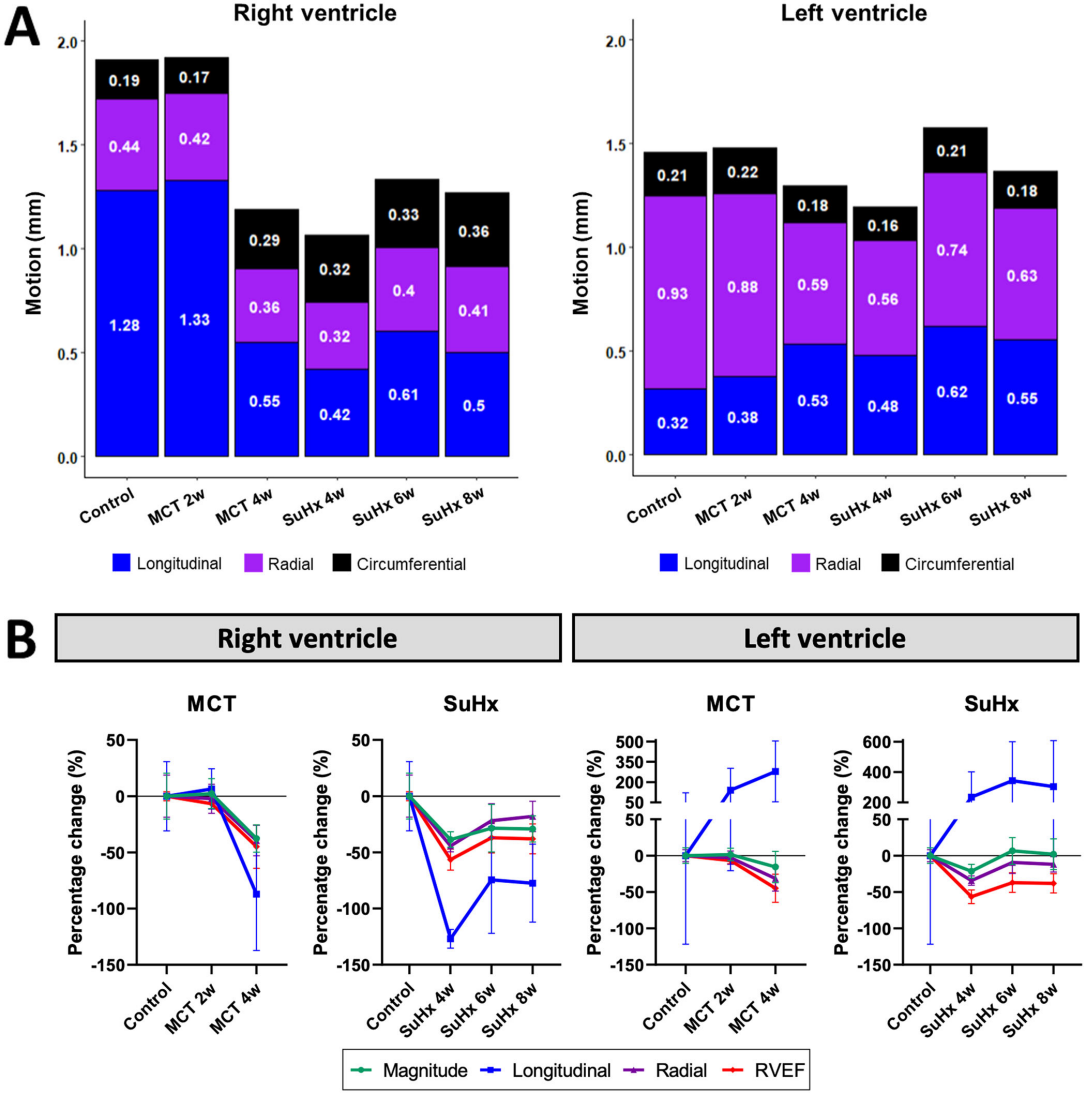
Regional cardiac motion derived from endocardial border movement between end‐diastole and end‐systole in rat models. (A) A stacked bar chart of right and left ventricular (RV, LV) magnitude of motion, depicted as a total sum of three motion components at control, monocrotaline (MCT; 4‐week) and Sugen hypoxia (SuHx; 4‐week) timepoints. Numbers within the bar chart show the corresponding average absolute motion of each component. (B) The percentage change of motion components and ejection fraction (EF) from average control values in MCT and SuHx rats. The percentage change was computed as the arithmetic mean of point‐wise percentage changes to account for spatial heterogeneity in ventricular wall motion, potentially resulting in large percentage changes.

Overall, the contribution of ventricular motion was markedly higher in the basal area in both animal models, indicating heterogeneous motion across the RV. A global decrease in RV wall motion was observed at maladapted disease stage in both animal models (Figure 5). In the MCT animals, the magnitude of RV wall motion was maintained at 2‐weeks and decreased by 37.7% (±12.2; *p* < 0.003) at 4‐weeks when compared to controls. In SuHx, the 4‐week timepoint demonstrated the greatest loss in motion (−38.6% ± 6.9%, *p* < 0.004), with subsequent mild recovery at 6‐ and 8‐weeks (−28.5% ± 21.1% from control, *p* < 0.03; and −29.2% ± 11.7% *p* < 0.05, respectively). Nevertheless, at all SuHx timepoints wall motion was decreased. The loss in RV motion was primarily attributed to the decline in longitudinal motion, followed by a decrease in radial motion at MCT 4‐weeks and SuHx 4‐weeks (Figure 6).

The left ventricular endocardial wall motion in control animals was less pronounced compared to that in the right ventricle. In the diseased timepoints, there were no differences in the average magnitude of motion yet proportional differences were observed between the radial and longitudinal components of motion (Figure 6). In the 4‐week MCT and 4‐week SuHx rats, radial motion was decreased significantly (−31.5% ± 17.2%, *p* < 0.01; and −34.4% ± 6.5%, *p* < 0.0003). While longitudinal motion was notably increased compared to the control group in both animal models, it reached significance only at SuHx 6‐week timepoint (*p* < 0.002).

**Figure 7:**
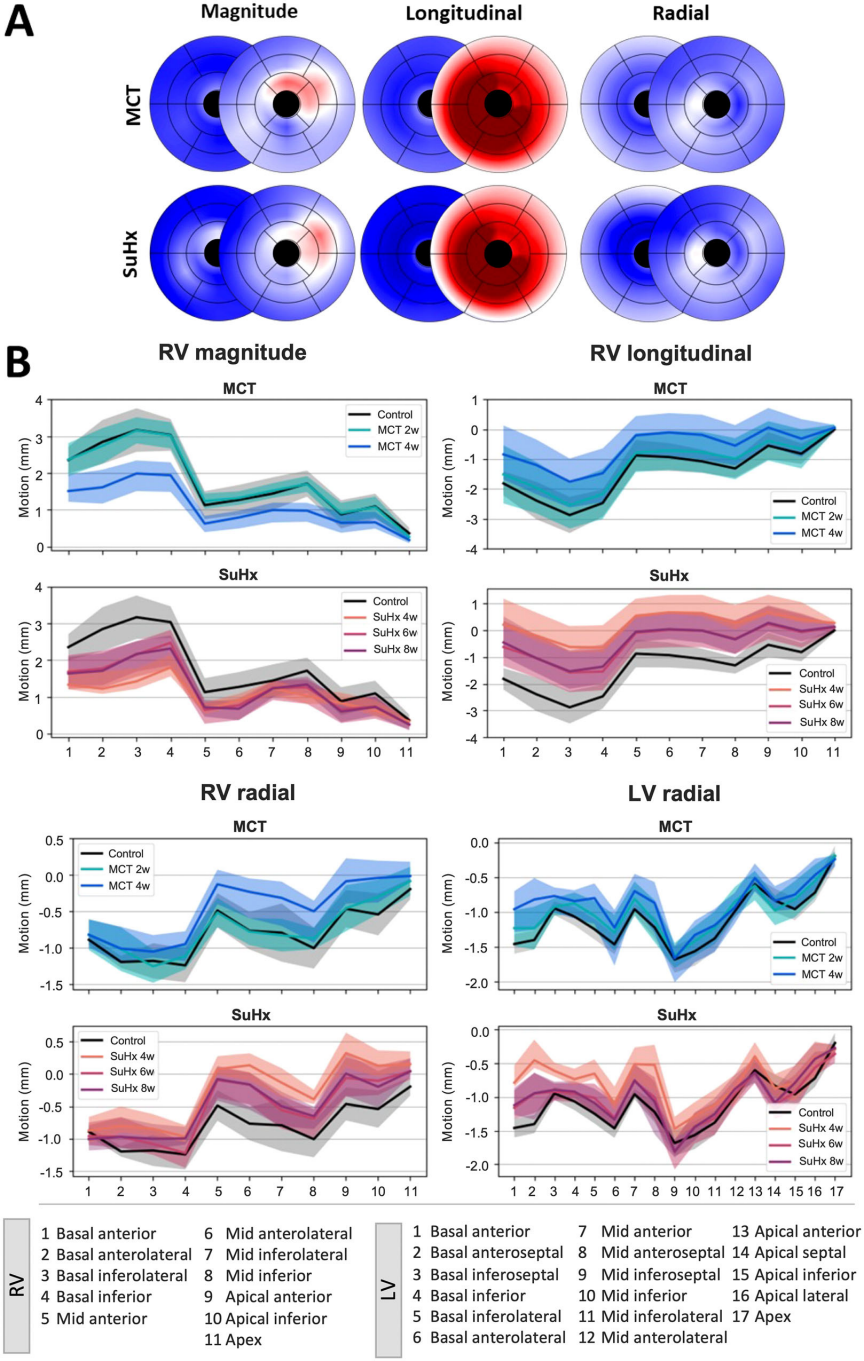
Regional cardiac motion from 28‐segment model displayed as a line graph for control, monocrotaline (MCT) and Sugen hypoxia (SuHx) timepoints. (A) The average point‐wise magnitude, longitudinal, and radial motion change measured as a percentage change from control for MCT and SuHx models, displayed on the 28‐segment model (17‐segments for the left, LV, and 11‐segments for the right ventricle, RV). The apical cap (segment 17 in LV and segment 11 in RV) was excluded. (B) The RV and LV motion components, which were statistically significant in differentiating in most regions between diseased and control groups, are shown. The bullseye regions are shown here numerically, with the corresponding regions defined in the bottom of the graph–left to right corresponds to basal to apical direction along the long‐axis of the ventricles.

The 28‐segment model enabled a standardized regional motion characterization (Figure 7). Specifically, the RV areas most indicative of disease progression were identified based on the extent of motion loss in the basal regions (Supplementary Table S4), the reduction of longitudinal motion from basal and mid‐ventricular regions (Supporting Information S1: Table S5), and the radial motion from the inferior and anterior apical regions (Supporting Information S1: Table S6). Interestingly, LV radial motion was also distinguishable between the diseased and control cohorts in the mid inferoseptal, inferior and inferolateral regions (Supporting Information S1: Table S7). The majority of differences were noted in the MCT 4‐week, SuHx 4‐week and SuHx 6‐week cohorts. It is important to note that areas with high relative changes (such as percentage change exceeding 100%) were due to notably low initial baseline values, where slight absolute differences between timepoints may seem excessively large in percentage terms. Absolute changes per region can be viewed in Supporting Information S1: Tables S3–S6.

## 4 Discussion

This study establishes a tool that produces time‐efficient, consistent, and accurate automated bi‐ventricular segmentations and generates 3D cardiac models from rat cardiac cine MR images. The inclusion of an atlas‐refinement step to produce high‐fidelity 3D cardiac meshes and whole ventricular motion mapping, has facilitated an in‐depth exploration into the regional phenotypes of cardiac function, thereby complementing the traditional cardiac MR indices such as end‐systolic volume and ejection fraction [19]. We utilized the two most established pulmonary hypertension rodent models, MCT and SuHx rats, to develop a deep learning‐based pipeline for the automated assessment of morphological variation and the evaluation of disease progression.

In the first stage of our pipeline, we developed and successfully validated the application of the fully convolutional network on rat cardiac MR images. We demonstrated the high accuracy of the automated segmentations of both LV and RV cavities and myocardium in healthy and PH‐induced rat ventricles. To date, recent literature on small animal ventricular segmentation has primarily been focused on the LV. For example, Huang et al. [20] as well as Fernández‐Llaneza et al. [1] tested multiple deep learning architectures for LV endocardial border segmentation in mice and rats, respectively. A mouse PH model‐based algorithm developed by Zufiria and colleagues is the only published preclinical segmentation network that includes RV labeling [12]. All three mentioned preclinical segmentation methods obtained high quality labels utilizing 3D networks either via 3D U‐Net [1, 2] or 3D attention M‐Net [20] architectures. Interestingly, despite using a 2D network in our study, we achieved comparable LV endocardial labeling performance and attained better results for bi‐ventricular segmentation than the previously reported Dice values in PH mice.

The interoperator variability analysis indicated that our trained FCN outperformed human annotators as it generated high quality and more consistent segmentations compared to manual segmentations across multiple human annotators. Firstly, the lower bias towards R1 and automated values compared to R1 and automated values were expected as only R1 datasets were used in model training. Secondly, the bias between inter‐operator variability closely mirrored the bias observed between R2 and automated values, indicating that the observed discrepancies were due to inter‐reader variability rather than model inaccuracy. Finally, the segmentation differences seen between the manual and automated segmentations were expected, as previous literature has identified (i) the right ventricular wall, (ii) the basal and apical slices, and (iii) the systolic phase as areas that present greater challenges for automated techniques from both patient [19, 21] and rodent studies [12]. The right ventricle is more complex in geometry regarding the overall shape, trabeculae intricacy [22], and notably thin myocardium. However, in our study, even the lowest discrepancy observed between the manual and automated RV myocardial wall labels resulted in an acceptable overall Dice value exceeding 0.83.

Inclusion of pathological states in training our model was crucial to ensure the applicability of the pipeline for future preclinical studies investigating disease mechanisms, progression, and treatments. Our trained FCN produced reliable automated segmentation aross groups and enabled the capture of disease progression and ventricular remodeling. However, the variability in estimating the right ventricular ejection fraction was higher for more severely pathological groups such as MCT 4‐weeks and SuHx 6‐week animal models, where the EF was below 50%, suggesting that segmentation quality should be visually controlled near end‐stage heart failure. Additionally, we demonstrated that our FCN was readily generalizable to unseen healthy and pathological female PH cohort, supporting its broader applicability on further animal models.

While assessing global cardiac function through EF is widely recognized as a sensitive indicator of pathology in both human and rat disease models, investigating the associated regional ventricular myocardial mechanics may offer an avenue for enhanced disease progression assessment [23, 24]. Regional bi‐ventricular wall analysis offers important and independent insights that are phenotypically or prognostically relevant in both healthy hearts [5] as well as diseases such as hypertrophic cardiomyopathy [24] and PAH [2]. Consequently, we developed a framework to assess these complex ventricular wall contraction patterns in the rat PH model from standard preclinical cardiac images. By incorporating an anatomical prior based shape‐refinement step, a high‐fidelity 3D cardiac mesh model could be generated.

The regional bi‐ventricular motion revealed a heterogeneous contractile pattern with basal motion predominance in healthy rat ventricles, driven primarily by the longitudinal motion, followed by radial movement. Our findings align with multiple sources from echocardiography and MRI studies performed on healthy volunteer human hearts [2, 25, 26, 27]. The contraction maps from our PH rat models corresponded to the motion patterns from RV remodeling in PH patients, where longitudinal strain was found as an early and prognostically valuable marker of RV dysfunction [28, 29]. The significant loss in right myocardial wall movement was primarily due to the decline in both basal longitudinal and apical radial components. These findings hold clinical relevance as both longitudinal and radial motions have been identified as the strongest predictors of survival in UK PAH patient populations [2, 23]. Furthermore, the observed motion pattern reinforces the concept of ventricular interdependence, where RV pressure overload can alter septal motion and reduce LV torsion, compounding biventricular dysfunction, as assessed by cardiac MRI feature tracking in a pediatric cohort and in rodents [29]. The consistency of these patterns across rodent models and human patients underscores the translational applicability of regional motion, strain and deformation analysis for assessing PH progression and associated mechanical remodeling.

Animal models mimicking disease progression and recapitulating important human prognostic factors are crucial to investigate disease initiation and progression [30], and as such, the longitudinal follow‐up via cardiac MR is becoming widely utilized in small animal models to study pathological heart remodeling non‐invasively. Our proposed method established in PH rodent disease models would further lend itself for studying other cardiac animal models that impact either the right or left ventricle, such as the heart failure with preserved ejection fraction model [31] or the aortic banding myocardial infarction model [3].

One of the limitations of this study is that the deep learning network was developed with scans acquired from our imaging center from ED and ES phases. We also noticed that severe ventricular remodeling at near end‐stage heart failure occasionally had an impact on the quality of the images, although this resulted in segmentation inaccuracies in only three cases. As such, a quality check is warranted for images that have low signal from the blood pools, which typically occurs near end‐stage heart failure. An additional limitation involves concerns regarding generalizability. We recommend that implementing the pipeline to new data from different scanners would require visual quality control, and potentially fine‐tuning via transfer learning with a subset of manual annotations. This process is important to ensure that the model handles variations in image resolution, contrast, and noise level of the scans. Given our model's excellent performance in segmenting left ventricular and right ventricular blood pool structures, we anticipate it will generalize well to other pathologies impacting the left ventricle or for estimating RV blood pool structural and functional measurements in RV‐specific conditions.

## 5 Conclusion

We presented a novel deep learning‐based algorithm that facilitates RV‐inclusive cardiac ventricular analysis of rodent imaging data and extraction of regional motion patterns from standard cine cardiac MR examinations. The proposed fully automated workflow is a suitable and fast method for producing cardiac segmentations in multiple animal models at various disease stages. The 3D models provided sensitive regional markers of disease progression and reaffirmed knowledge of ventricular contractile patterns of the control and PH rat models that are in keeping with data from PH patients. Importantly, this automated bi‐ventricular rat‐specific pipeline is in alignment with clinical cardiac imaging AI developments, serving as a valuable resource to translate outcomes from preclinical studies to clinical findings. It is anticipated that, the ease of segmentation and the process of obtaining cardiac indices coupled with novel motion phenotypic trait representations will (i) encourage future preclinical studies to incorporate cardiac noninvasive imaging techniques and (ii) stimulate future research into similar assistive methods and workflows.

## Author Contributions

Marili Niglas, Nicoleta Baxan and Lin Zhao drafted the manuscript. Jinming Duan, Declan O'Regan, Timothy JW Dawes and Wenjia Bai established the DL model pipeline for data augmentation, training. Timothy JW Dawes established pipelines for mesh‐based motion mapping. Marili Niglas, Ali Ashek, Chen Nien‐Chen and Chongyang Xie established the animal models. Nicoleta Baxan established the MRI protocols. Marili Niglas, Ali Ashek, Lin Zhao, Nicoleta Baxan anaesthetized and monitored the animals during MRI examinations. Marili Niglas and Nicoleta Baxan carried out the manual segmentation of MR images. Marili Niglas performed the DL model training and validation, statistical analysis, and designing the figures and tables. Lan Zhao, Nicoleta Baxan and Wenjia Bai supervised the study and discussed the study concept and results. All authors reviewed the final manuscript.

## Ethics Statement

All animal procedures were reviewed and approved by the Imperial College Animal Welfare and Ethical Review Body. The authors complied with the ARRIVE guidelines.

## Conflicts of Interest

The authors declare no conflicts of interest.

## Supporting information

Supplementary.docx.

## Data Availability

Materials, data, and associated protocols will be available to readers upon request after the manuscript is accepted.
