# Supplementary material for: Automated Bi‐Ventricular Segmentation and Regional Cardiac Wall Motion Analysis for Rat Models of Pulmonary Hypertension
Source: Pulm Circ. 2025 May 12;15(2):e70092. doi: 10.1002/pul2.70092 (PMC12067408; doi:10.1002/pul2.70092)
Supplement: Supplementary file 1 — Supplementary.docx. [file PUL2-15-e70092-s001.docx]

**Supplementary figures**


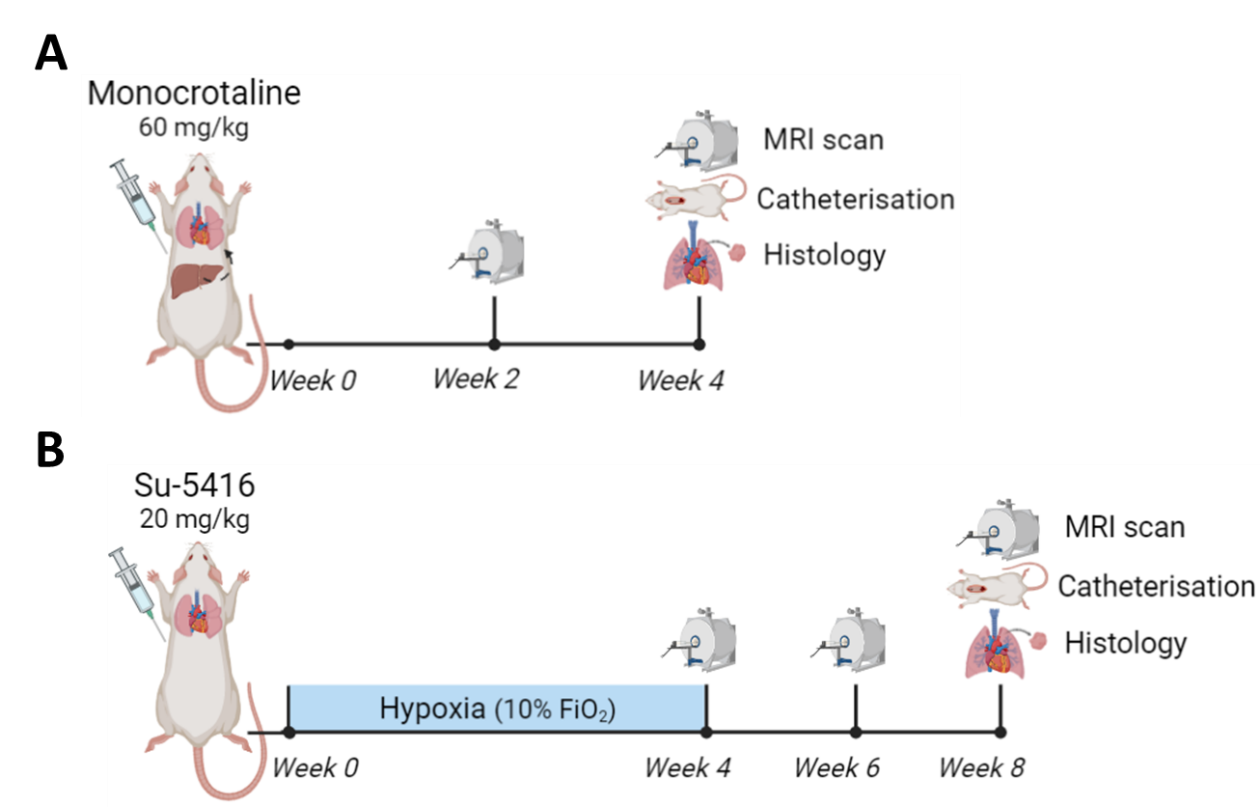


**Supplementary figure S1. Pulmonary hypertension animal model establishment.** A - Monocrotaline (MCT) animal model was established via subcutaneous MCT (60mg/ml) injection. Non-invasive magnetic resonance imaging (MRI) was conducted until the final endpoint of 4-weeks post injection, where catheterisation measurements and tissues for histological analysis were obtained. B - Sugen hypoxia (SuHx) model was derived by, firstly, Sugen (20mg/ml) injection, followed by 4 weeks of hypoxia (FiO2 0.1) exposure in a normobaric chamber. Non-invasive imagine was performed along with invasive procedures at the endpoint of 8 weeks post injection.


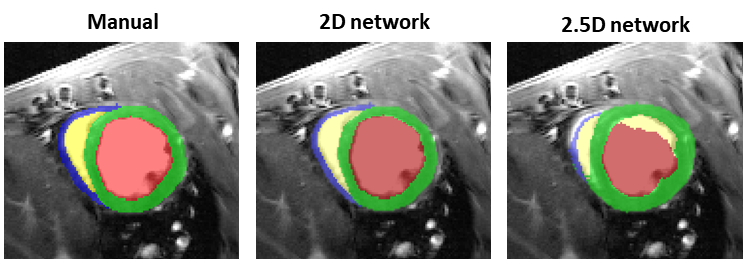


**Supplementary figure S2. Visual comparison of rat segmentations from manual, 2D fully convolutional network (FCN), and 2.5D FCN produced models.** Red – Left ventricular blood pool; green – left ventricular myocardial wall; yellow – right ventricular blood pool; blue – right ventricular myocardial wall. The networks were trained on the same rodent training datasets. The 2D network processed each slice independently, while the 2.5D network used adjacent slices as additional input channels to incorporate z-stacked (longitudinal) context. Visual errors of the 2.5D network segmentations included undersegmentation of the right ventricular wall and mislabelled left ventricular cavity.

**Supplementary table S1. Data augmentation table. Details of the data augmentation type along with corresponding random distributions and ranges of the parameters applied.**

| Augmentation type | Randomness source | Distribution | Set Parameters | Notes |
| --- | --- | --- | --- | --- |
| Shift | np.random.normal() | Clipped normal | ±30 pixels | Applied independently on x and y axes |
| Rotation | np.random.normal and np.random.uniform() | Clipped normal and uniform | ±30° around 0 | Random base of 0° |
| Intensity | np.random.normal() | Clipped normal | ± 0.3 | Multiply pixel values |


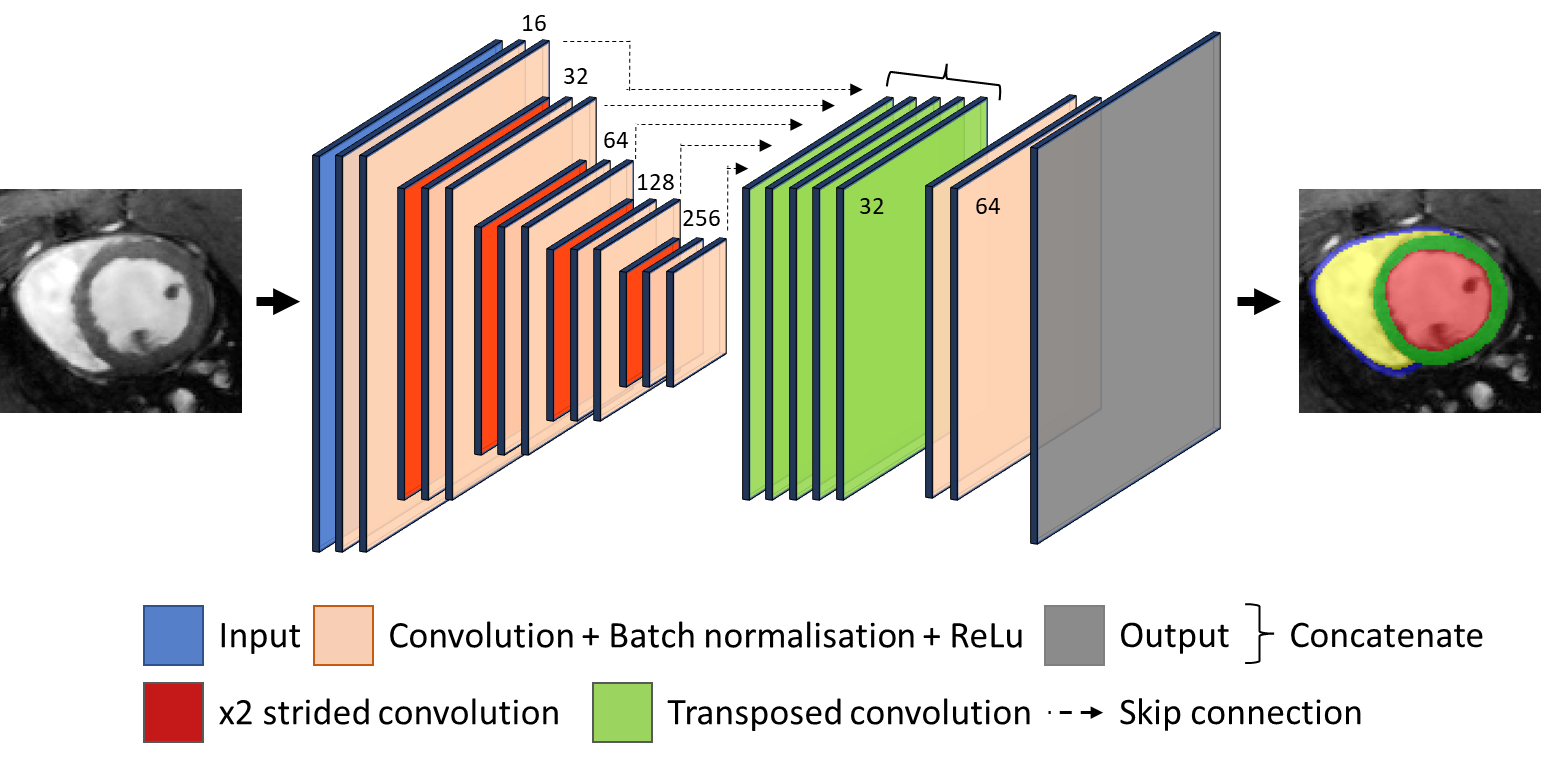


**Supplementary figure S3. Fully convolutional network architecture.** The cine images with corresponding manual segmentations are used to train the fully convolutional network (FCN). As the network takes volumetric labels as inputs, the data augmentation was done by intensity rescaling and affine transformations, including isotropic scaling and rotation*.* The FCN is comprised of 15 convolutional layers with transposed layers and skip connections to retain the course to fine details. The network uses softmax cross-entropy loss which is minimised by the stochastic gradient descent in back-propagation, trained over 200 epochs. The trained model was deployed on unseen test set data, creating an automated segmentation. Number of atlases included in the training range from *n_i_* to *n_i_+i*. Number of test set images range from *n_x_* to *n_x_+x*.


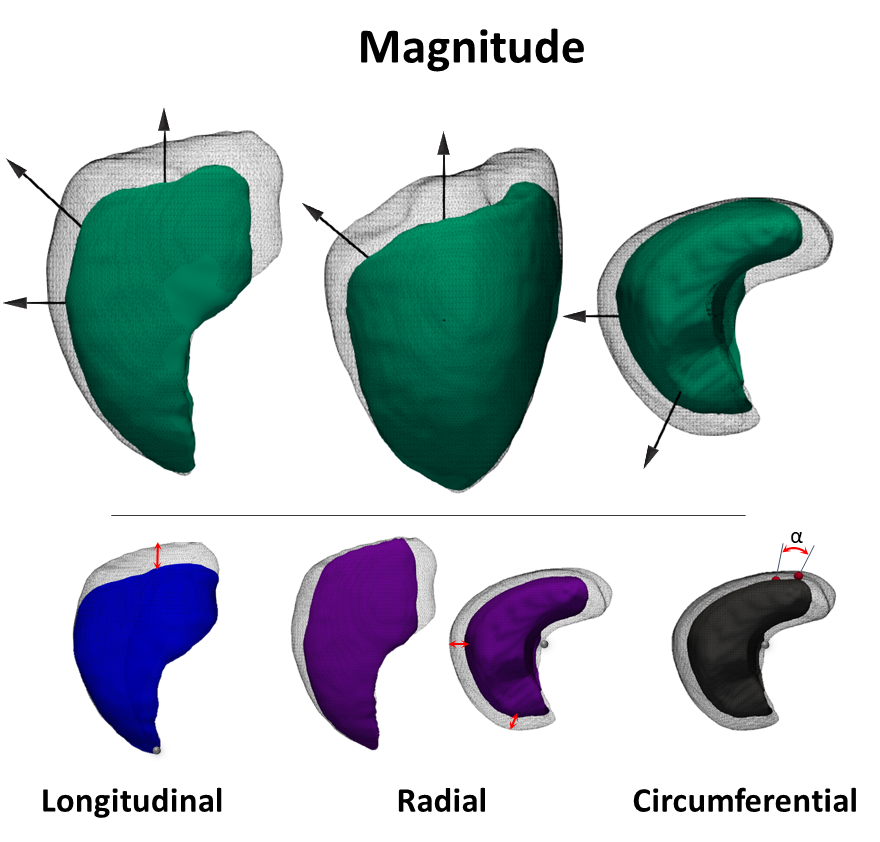


**Supplementary figure S4. 3D right ventricular cardiac motion and wall motion components illustratrated on meshes.** *Top* – Magnitude of motion display with the end-diastolic (ED) phase depicted as a black wired mesh, and the end-systolic (ES) phase depicted as a green solid mesh. Movement was calculated in all three directions as a composite of three ventricular endocardial wall motino components. *Bottom* – Longitudinal, radial and circumferential walls depiced at ED phase as a black wired mesh and at the ES phase as a solid mesh (blue, purple and black, respectively). Longitudinal wall motion was calculated as the distance between two corresponding points from the longitudinal (z-axis) direction between ED and ES phases; radial as the distance between the ED and ES points in relation to a centreline (from apex-to-base at the septal wall; shown as a grey dot); and circumferential motion calculated as angular distance travelled between two corresponding points from ED to ES.


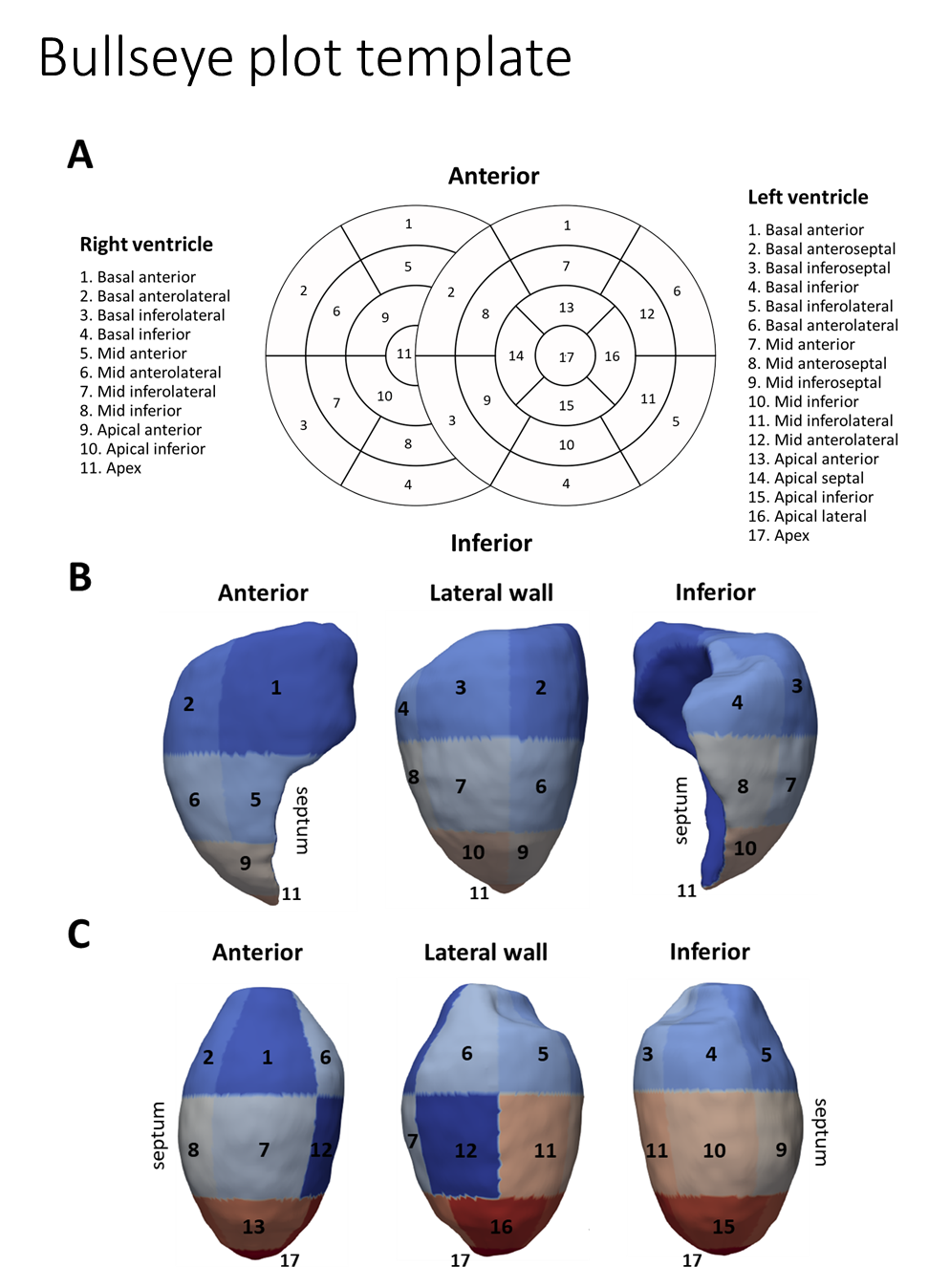


**Supplementary figure S5. 28-segment bullseye plot template and corresponding mesh.** *A* – The 28-segment bullseye plot template shows basal to apical segments that were taken longitudinally, flattened in a short-axis view. A custom-defined regions from the left ventricle (17-segments) and right ventricle (11-segments) are displayed with corresponding numeration and regional names. *B* – A custom-made right ventricular 11-segment model displayed on a 3D mesh with corresponding numeration. *C* – A custom-made left ventricular 17-segment model displayed on a 3D mesh with respective numeration.

**
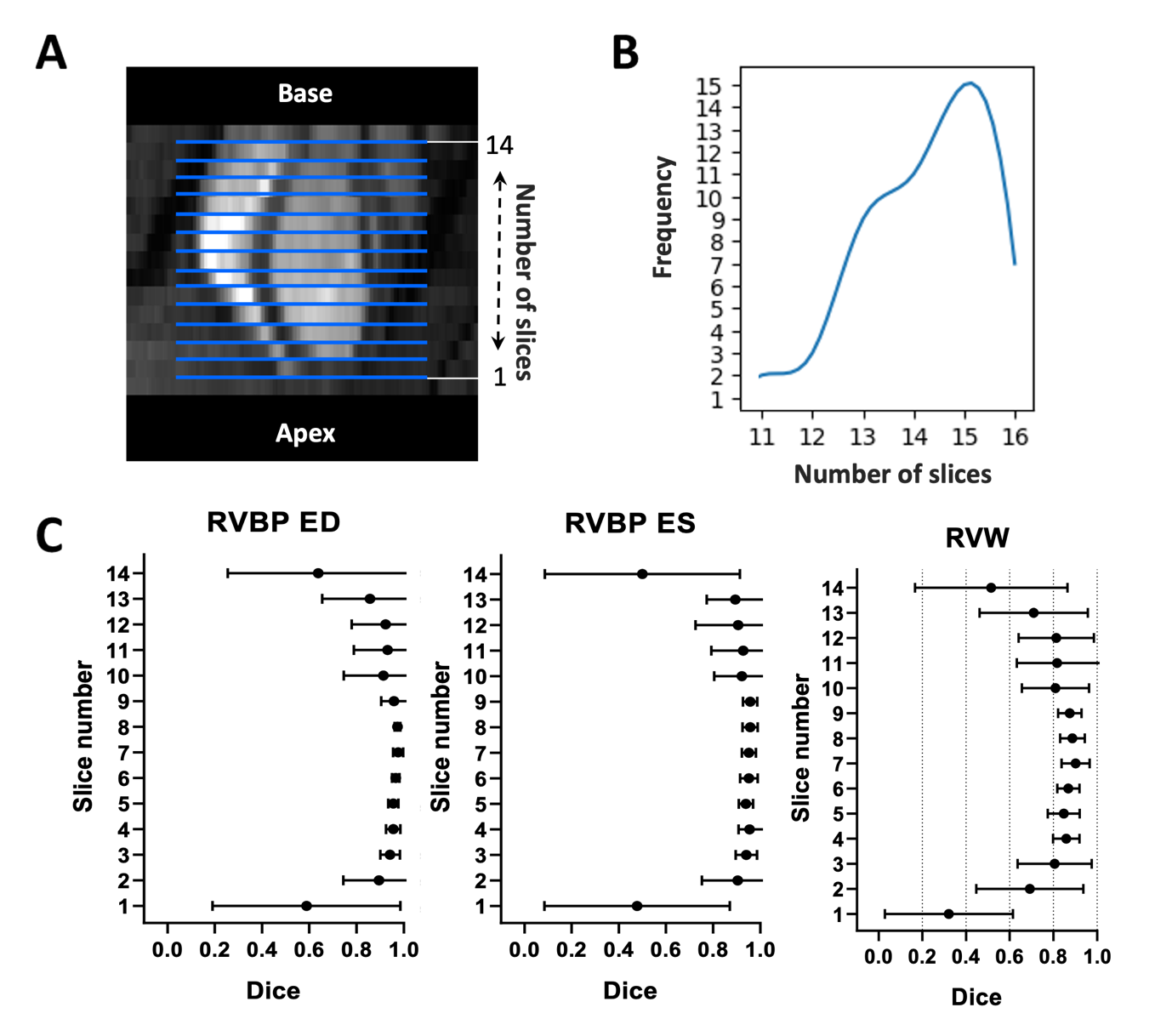
**

**Supplementary figure S6.** **Slice-wise comparison of Dice correlation coefficient.** A – A representative image of slice coverage – slice numbers starting from apex to base (number 1 to 14). B – Absolute frequency distribution of the number of slices in our test datasets. The varying number of slices was due to the method of CMR acquisition – the cine planning accounted for bi-ventricular coverage based on specific landmarks, not on slice thickness or number. C – The Dice correlation coefficient graphs for end-diastolic (ED; left), end-systolic (ES; right) phases for right ventricular blood pool (RVBP), and right ventricular wall (RVW) from manual and automated CINE segmentations at each slice through the ventricles. Firstly, slice-wise Dice correlation coefficient was calculated (a Dice value for each slice within the one dataset). Secondly, the array of Dice correlation coefficient values per slices was split into 14 proportions to have an approximate matching between anatomically coherent slices.


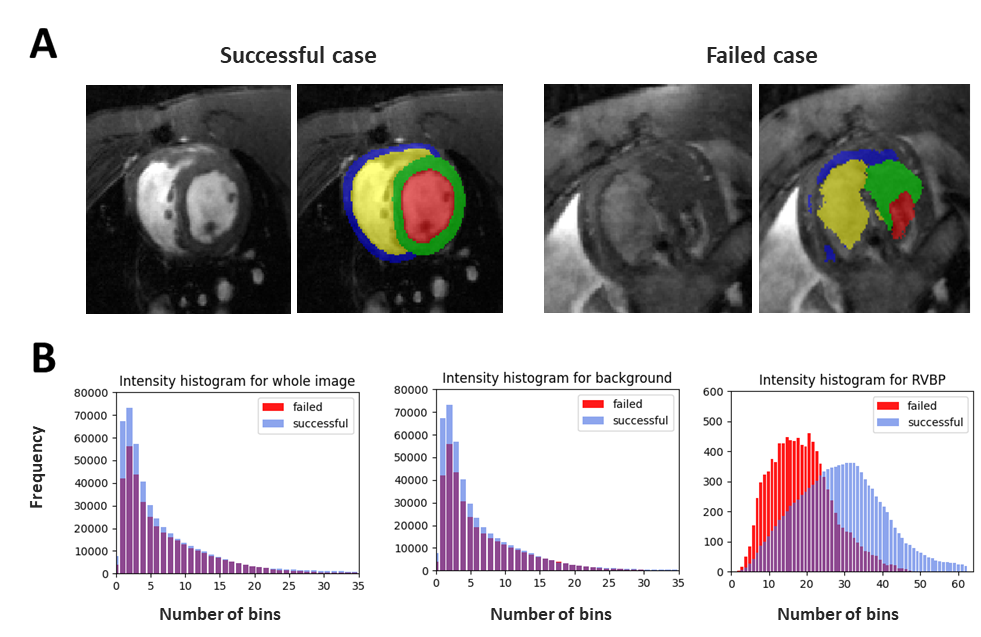


**Supplementary figure S7. Comparison of successful and failed segmentations.** A – Visual comparison between the images and automatically generated segmentations of a successfully segmented case (healthy subject) and a failed one (MCT 4-week). B – Frequency histograms for pixel intensities of all of the successfully segmented cases (red, n=44) and failed cases (blue, n=3). The intensities were derived from whole images (left), background labels (middle) and right ventricular blood pool cavities (RVBP; right).

**Supplementary table S2. Statistical information from Bland-Altman plots from automated method validation on test set.** The bias ± standard deviation and corresponding 95% upper and lower confidence interval (CI) limits of agreement are reported.

| **Cardiac indices** |  | **Bias** | **95% CI  upper limit** | **95% CI  lower limit** |
| --- | --- | --- | --- | --- |
| RVEDV | µl | 7.94 ± 23.27 | -37.66 | 53.54 |
|  | % | 1.18 ± 3.33 | -5.35 | 7.71 |
| RVESV | µl | 4.73 ± 19.13 | -32.77 | 42.24 |
|  | % | 1.26 ± 4.61 | -7.78 | 10.3 |
| RVSV | µl | 3.23 ± 19.76 | -35.50 | 41.96 |
|  | % | 1.04 ± 8.31 | -15.27 | 17.34 |
| RVEF | % | -0.023 ± 2.31 | -4.55 | 4.50 |
|  | % | -0.15 ± 6.87 | -13.62 | 13.31 |
| RVM | mg | -34.14 ± 22.95 | -79.12 | 10.83 |
|  | % | -10.25 ± 6.74 | -23.47 | 2.96 |
| LVEDV | µl | -0.99 ± 12.26 | -25.03 | 23.03 |
|  | % | -0.34 ± 3.07 | -6.37 | 5.68 |
| LVESV | µl | 0.51 ± 9.92 | -18.93 | 19.94 |
|  | % | 0.11 ± 5.47 | -10.60 | 10.82 |
| LVSV | µl | 1.61 ± 14.06 | -29.17 | 25.95 |
|  | % | -0.58 ± 5.92 | -12.17 | 11.02 |
| LVEF | % | -0.14 ± 2.31 | -4.66 | 4.38 |
|  | % | -0.19 ± 4.04 | -8.11 | 7.74 |
| LVM | mg | -19.40 ± 24.11 | -66.66 | 27.85 |
|  | % | -3.67 ± 4.68 | -12.85 | 5.50 |


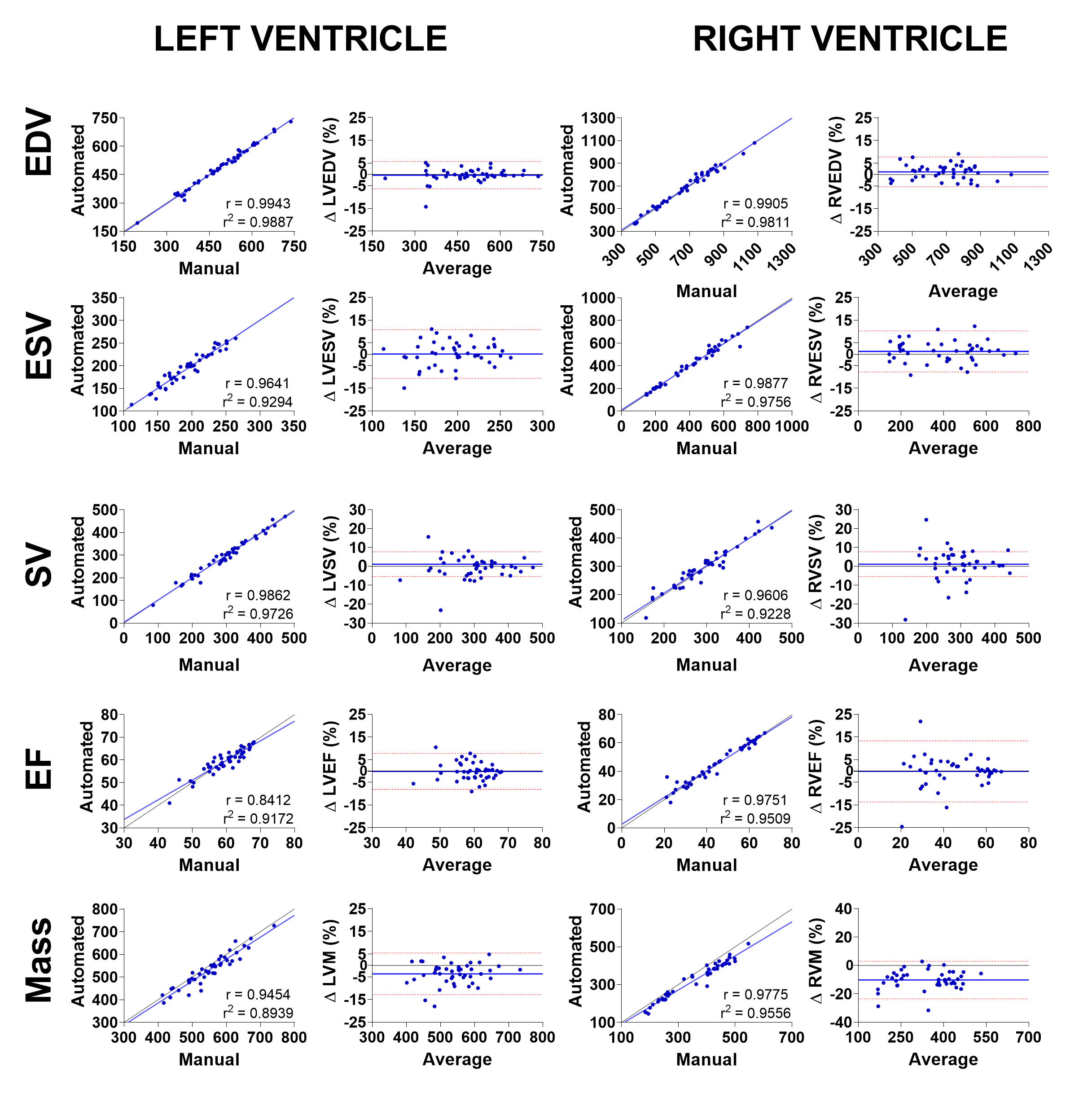


**Supplementary figure S8. Interoperator variability assessment via Blan-Altman plots.** Reader one (R1) manual segmentation based cardiac parametres like right ventricular (RV) and left ventricular (LV) end-diastolic volume (EDV), end-systolic volume (ESV), stroke volume (SV), mass (Mass) and ejection fraction (EF) were compared against automated method (Auto) and reader two (R2) derived segmentation values. The equality line is marked as a continuous black line, and the bias line is indicated as the continuous blue line. The limits of agreement are marked with interrupted red lines.

**Supplementary table S3. Statistical information from Bland-Altman plots from interoperator agreement study.** For each right ventricular cardiac parameter, the reader one and automated segmentation, and reader one and reader two segmentation comparison via Bland-Altman is shown. The bias ± standard deviation and corresponding 95% upper and lower confidence interval (CI) limits of agreement are reported.

| **Cardiac parameter** | **Method** | **Bias** | **SD of bias** | **95% CI  upper limit** | **95% CI  lower limit** | **Limits of agreement range** |
| --- | --- | --- | --- | --- | --- | --- |
| RVEDV (µl) | R1 v R2 | 34.2 | 28.59 | -21.80 | 90.26 | 112.06 |
|  | Auto v R1 | 5.68 | 23.85 | -41.07 | 52.43 | 93.5 |
|  | Auto v R2 | -28.6 | 24.50 | -76.57 | 19.46 | 96.03 |
| RVESV (µl) | R1 v R2 | 37.1 | 26.86 | -15.50 | 89.78 | 105.28 |
|  | Auto v R1 | -0.55 | 28.29 | -56.00 | 54.89 | 110.89 |
|  | Auto v R2 | -37.7 | 29.21 | -94.21 | 19.55 | 113.76 |
| RVEF (%) | R1 v R2 | -2.58 | 3.84 | -10.10 | 4.94 | 15.04 |
|  | Auto v R1 | 0.75 | 3.74 | -6.57 | 8.08 | 14.65 |
|  | Auto v R2 | 3.33 | 4.10 | -4.71 | 11.37 | 16.08 |
| RVM (mg) | R1 v R2 | -6.44 | 23.71 | -52.91 | 40.02 | 92.93 |
|  | Auto v R1 | -33.2 | 18.45 | -69.38 | 2.92 | 72.3 |
|  | Auto v R2 | -26.8 | 30.7 | -87.00 | 33.39 | 120.39 |


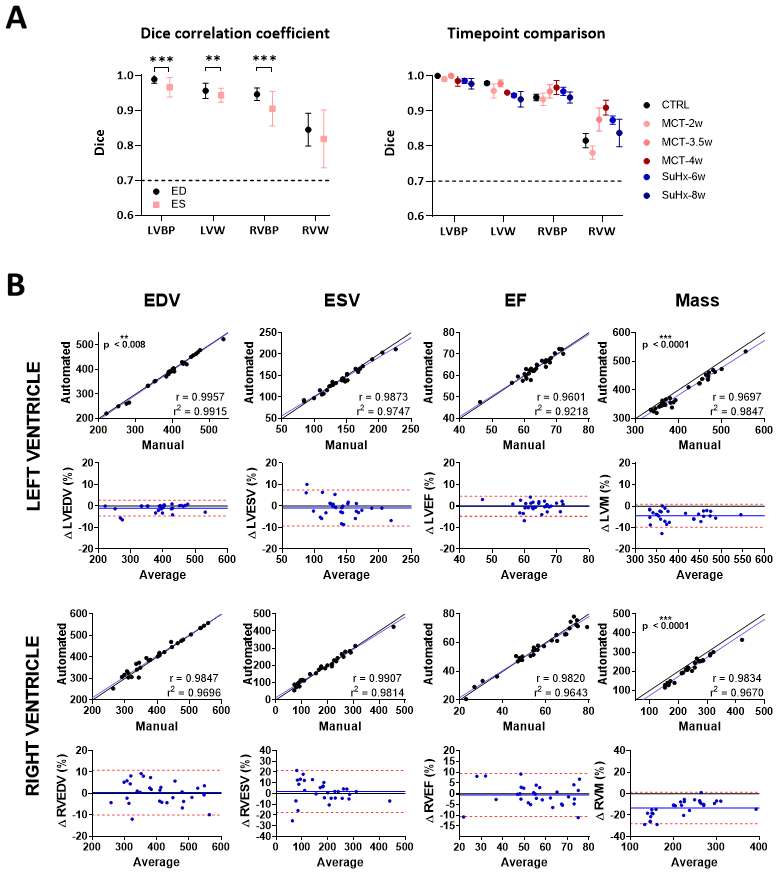


**Supplementary figure S9. Model evaluation on an unseen dataset of female control, monocrotaline (MCT) and Sugen hypoxia (SuHx) rat models.** A – Dice correlation coefficient and Hausdorff distance comparison between labels at end-diastole (ED) and end-systole (ES). B – Groupwise comparisons were made between Dice coefficient metric labels between each timepoint. Template and one to seven best atlas (BA) registration method average Dice values and the corresponding standard deviations (SD) are given for each label. The labels included right ventricle (RV) and left ventricular (LV) cavity and wall – and each cardiac phase segmented – end-diastolic and end-systolic. Best results are highlighted in bold.

**Supplementary table S4. Right ventricular magnitude of wall motion from control, monocrotaline (MCT) and Sugen hypoxia (SuHx) timepoints from 17 regions from the endocardial wall motion.** Significance values are as follows: * p<0.05, ** p<0.01, *** p<0.001, **** p<0.0001.

| Regions | Control | | | MCT 2w | | | | MCT 4w | | | | SuHx 4w | | | | SuHx 6w | | | | SuHx 8w | | | |
| --- | --- | --- | --- | --- | --- | --- | --- | --- | --- | --- | --- | --- | --- | --- | --- | --- | --- | --- | --- | --- | --- | --- | --- |
| basal anterior | 2.35 | ± | 0.36 | 2.36 | ± | 0.45 |  | 1.51 | ± | 0.29 | ** | 1.33 | ± | 0.13 | *** | 1.68 | ± | 0.47 | ** | 1.63 | ± | 0.33 | * |
| basal anterolateral | 2.84 | ± | 0.60 | 2.74 | ± | 0.48 |  | 1.61 | ± | 0.44 | ** | 1.22 | ± | 0.14 | *** | 1.77 | ± | 0.52 | ** | 1.71 | ± | 0.39 | * |
| basal inferolateral | 3.17 | ± | 0.60 | 3.16 | ± | 0.36 |  | 1.99 | ± | 0.48 | ** | 1.42 | ± | 0.21 | *** | 2.15 | ± | 0.61 | ** | 2.17 | ± | 0.48 |  |
| basal inferior | 3.03 | ± | 0.43 | 3.02 | ± | 0.35 |  | 1.94 | ± | 0.45 | ** | 1.80 | ± | 0.25 | *** | 2.47 | ± | 0.70 |  | 2.31 | ± | 0.46 |  |
| mid anterior | 1.13 | ± | 0.38 | 1.23 | ± | 0.21 |  | 0.62 | ± | 0.23 | * | 0.64 | ± | 0.12 | * | 0.64 | ± | 0.38 | * | 0.71 | ± | 0.25 |  |
| mid anterolateral | 1.27 | ± | 0.41 | 1.32 | ± | 0.18 |  | 0.79 | ± | 0.30 |  | 0.90 | ± | 0.12 |  | 0.77 | ± | 0.40 |  | 0.67 | ± | 0.31 |  |
| mid inferolateral | 1.44 | ± | 0.44 | 1.52 | ± | 0.20 |  | 0.99 | ± | 0.34 |  | 1.23 | ± | 0.21 |  | 1.24 | ± | 0.34 |  | 1.23 | ± | 0.24 |  |
| mid inferior | 1.71 | ± | 0.36 | 1.70 | ± | 0.22 |  | 0.98 | ± | 0.30 | ** | 1.02 | ± | 0.21 | ** | 1.26 | ± | 0.45 |  | 1.34 | ± | 0.24 |  |
| apical anterior | 0.88 | ± | 0.38 | 0.91 | ± | 0.28 |  | 0.64 | ± | 0.27 |  | 0.83 | ± | 0.05 |  | 0.66 | ± | 0.38 |  | 0.60 | ± | 0.29 |  |
| apical inferior | 1.09 | ± | 0.35 | 1.07 | ± | 0.28 |  | 0.66 | ± | 0.18 | * | 0.62 | ± | 0.16 | * | 0.73 | ± | 0.33 |  | 0.72 | ± | 0.23 |  |
| apex | 0.36 | ± | 0.15 | 0.26 | ± | 0.09 |  | 0.18 | ± | 0.09 |  | 0.33 | ± | 0.04 |  | 0.24 | ± | 0.13 |  | 0.24 | ± | 0.17 |  |

**Supplementary table S5. Right ventricular longitudinal wall motion from control, monocrotaline (MCT) and Sugen hypoxia (SuHx) timepoints from 17 regions from the endocardial wall motion.** Significance values are as follows: * p<0.05, ** p<0.01, *** p<0.001, **** p<0.0001.

| Regions | Control | | | MCT 2w | | | | MCT 4w | | | | SuHx 4w | | | | SuHx 6w | | | | SuHx 8w | | | |
| --- | --- | --- | --- | --- | --- | --- | --- | --- | --- | --- | --- | --- | --- | --- | --- | --- | --- | --- | --- | --- | --- | --- | --- |
| basal anterior | -1.82 | ± | 0.40 | -1.51 | ± | 0.97 |  | -0.84 | ± | 0.84 | * | 0.20 | ± | 0.09 | **** | -0.63 | ± | 0.71 | *** | -0.46 | ± | 0.52 | * |
| basal anterolateral | -2.40 | ± | 0.63 | -1.96 | ± | 0.86 |  | -1.20 | ± | 0.98 | ** | -0.21 | ± | 0.20 | **** | -1.04 | ± | 0.74 | ** | -1.05 | ± | 0.55 | * |
| basal inferolateral | -2.88 | ± | 0.61 | -2.55 | ± | 0.78 |  | -1.76 | ± | 0.87 | * | -0.63 | ± | 0.19 | **** | -1.58 | ± | 0.74 | ** | -1.54 | ± | 0.66 |  |
| basal inferior | -2.48 | ± | 0.45 | -2.17 | ± | 0.83 |  | -1.48 | ± | 0.97 | * | -0.65 | ± | 0.19 | **** | -1.55 | ± | 0.70 | ** | -1.37 | ± | 0.63 |  |
| mid anterior | -0.88 | ± | 0.51 | -0.79 | ± | 0.65 |  | -0.21 | ± | 0.71 | * | 0.53 | ± | 0.10 | *** | -0.10 | ± | 0.59 | * | -0.06 | ± | 0.38 |  |
| mid anterolateral | -0.93 | ± | 0.46 | -0.71 | ± | 0.65 |  | -0.11 | ± | 0.79 | * | 0.66 | ± | 0.05 | *** | 0.01 | ± | 0.65 | ** | 0.03 | ± | 0.37 | * |
| mid inferolateral | -1.08 | ± | 0.39 | -0.77 | ± | 0.68 |  | -0.19 | ± | 0.83 | * | 0.64 | ± | 0.13 | **** | -0.01 | ± | 0.63 | *** | -0.02 | ± | 0.45 | * |
| mid inferior | -1.32 | ± | 0.31 | -0.99 | ± | 0.69 |  | -0.55 | ± | 0.67 | * | 0.25 | ± | 0.23 | **** | -0.32 | ± | 0.57 | *** | -0.35 | ± | 0.54 |  |
| apical anterior | -0.55 | ± | 0.46 | -0.39 | ± | 0.66 |  | 0.06 | ± | 0.61 |  | 0.66 | ± | 0.08 | *** | 0.23 | ± | 0.56 | ** | 0.27 | ± | 0.33 | * |
| apical inferior | -0.82 | ± | 0.34 | -0.66 | ± | 0.66 |  | -0.32 | ± | 0.61 |  | 0.39 | ± | 0.11 | **** | -0.08 | ± | 0.58 | ** | -0.03 | ± | 0.38 |  |
| apex | -0.01 | ± | 0.09 | 0.06 | ± | 0.10 |  | 0.05 | ± | 0.11 |  | 0.26 | ± | 0.04 | *** | 0.11 | ± | 0.16 |  | 0.12 | ± | 0.21 |  |

**Supplementary table S6. Right ventricular radial wall motion from control, monocrotaline (MCT) and Sugen hypoxia (SuHx) timepoints from 17 regions from the endocardial wall motion.** Significance values are as follows: * p<0.05, ** p<0.01, *** p<0.001, **** p<0.0001.

| Regions | Control | | | MCT 2w | | | | MCT 4w | | | | | SuHx 4w | | | | | SuHx 6w | | | | SuHx 8w | | | |
| --- | --- | --- | --- | --- | --- | --- | --- | --- | --- | --- | --- | --- | --- | --- | --- | --- | --- | --- | --- | --- | --- | --- | --- | --- | --- |
| basal anterior | -0.89 | ± | 0.07 | -0.82 | ± | 0.21 |  | -0.82 | ± | 0.18 |  | -0.87 | | ± | 0.19 |  | -0.96 | | ± | 0.14 |  | -1.01 | ± | 0.18 |  |
| basal anterolateral | -1.20 | ± | 0.08 | -1.02 | ± | 0.30 |  | -1.01 | ± | 0.20 | * | -0.81 | | ± | 0.10 | ** | -0.97 | | ± | 0.17 | ** | -0.97 | ± | 0.18 |  |
| basal inferolateral | -1.18 | ± | 0.24 | -1.26 | ± | 0.23 |  | -1.05 | ± | 0.08 |  | -0.85 | | ± | 0.11 | * | -1.08 | | ± | 0.17 |  | -1.00 | ± | 0.15 |  |
| basal inferior | -1.24 | ± | 0.23 | -1.12 | ± | 0.18 |  | -0.95 | ± | 0.23 | * | -0.97 | | ± | 0.09 |  | -1.22 | | ± | 0.23 |  | -1.00 | ± | 0.09 |  |
| mid anterior | -0.49 | ± | 0.24 | -0.52 | ± | 0.20 |  | -0.13 | ± | 0.27 |  | 0.07 | | ± | 0.09 | *** | -0.09 | | ± | 0.27 | ** | -0.08 | ± | 0.33 |  |
| mid anterolateral | -0.77 | ± | 0.26 | -0.78 | ± | 0.19 |  | -0.23 | ± | 0.36 |  | 0.13 | | ± | 0.24 | ** | -0.16 | | ± | 0.31 | *** | -0.16 | ± | 0.39 |  |
| mid inferolateral | -0.79 | ± | 0.40 | -0.83 | ± | 0.23 |  | -0.32 | ± | 0.39 |  | -0.14 | | ± | 0.22 | * | -0.56 | | ± | 0.29 |  | -0.50 | ± | 0.31 |  |
| mid inferior | -1.01 | ± | 0.28 | -0.87 | ± | 0.15 |  | -0.50 | ± | 0.43 |  | -0.39 | | ± | 0.07 | ** | -0.69 | | ± | 0.29 |  | -0.65 | ± | 0.20 |  |
| apical anterior | -0.46 | ± | 0.25 | -0.46 | ± | 0.32 |  | -0.09 | ± | 0.31 | * | 0.32 | | ± | 0.22 | ** | -0.06 | | ± | 0.29 | * | 0.01 | ± | 0.35 |  |
| apical inferior | -0.54 | ± | 0.29 | -0.30 | ± | 0.24 |  | -0.04 | ± | 0.34 | ** | 0.13 | | ± | 0.17 | ** | -0.11 | | ± | 0.22 | * | -0.20 | ± | 0.32 |  |
| apex | -0.20 | ± | 0.14 | -0.09 | ± | 0.20 |  | -0.02 | ± | 0.08 | * | 0.15 | | ± | 0.06 | *** | 0.04 | | ± | 0.12 | ** | 0.04 | ± | 0.08 | * |

**Supplementary table S7. Left ventricular radial wall motion from control, monocrotaline (MCT) and Sugen hypoxia (SuHx) timepoints from 17 regions from the endocardial wall motion.** Significance values are as follows: * p<0.05, ** p<0.01, *** p<0.001, **** p<0.0001.

| Regions | Control | | | MCT 2w | | | | MCT 4w | | | | SuHx 4w | | | | SuHx 6w | | | | SuHx 8w | | | |
| --- | --- | --- | --- | --- | --- | --- | --- | --- | --- | --- | --- | --- | --- | --- | --- | --- | --- | --- | --- | --- | --- | --- | --- |
| basal anterior | -1.46 | ± | 0.14 | -1.23 | ± | 0.26 |  | -0.96 | ± | 0.33 | *** | -0.79 | ± | 0.06 | **** | -1.16 | ± | 0.16 | ** | -1.12 | ± | 0.14 | * |
| basal anteroseptal | -1.40 | ± | 0.14 | -1.23 | ± | 0.31 |  | -0.82 | ± | 0.44 | ** | -0.46 | ± | 0.11 | **** | -0.95 | ± | 0.29 | *** | -0.95 | ± | 0.39 |  |
| basal inferoseptal | -0.95 | ± | 0.10 | -0.93 | ± | 0.12 |  | -0.77 | ± | 0.24 |  | -0.61 | ± | 0.08 | ** | -0.92 | ± | 0.13 |  | -0.89 | ± | 0.14 |  |
| basal inferior | -1.07 | ± | 0.13 | -0.88 | ± | 0.16 |  | -0.85 | ± | 0.25 |  | -0.74 | ± | 0.09 | ** | -0.95 | ± | 0.13 |  | -0.92 | ± | 0.09 |  |
| basal inferolateral | -1.25 | ± | 0.15 | -1.05 | ± | 0.21 |  | -0.80 | ± | 0.37 | * | -0.65 | ± | 0.08 | **** | -1.01 | ± | 0.23 | * | -1.09 | ± | 0.16 |  |
| basal anterolateral | -1.47 | ± | 0.14 | -1.30 | ± | 0.26 |  | -1.23 | ± | 0.55 |  | -1.10 | ± | 0.11 | ** | -1.31 | ± | 0.17 |  | -1.33 | ± | 0.11 |  |
| mid anterior | -0.96 | ± | 0.08 | -0.81 | ± | 0.25 |  | -0.70 | ± | 0.22 | * | -0.51 | ± | 0.23 |  | -0.77 | ± | 0.19 | * | -0.75 | ± | 0.21 |  |
| mid anteroseptal | -1.22 | ± | 0.15 | -1.13 | ± | 0.30 |  | -0.87 | ± | 0.43 | ** | -0.53 | ± | 0.42 |  | -1.01 | ± | 0.33 |  | -1.07 | ± | 0.48 |  |
| mid inferoseptal | -1.68 | ± | 0.19 | -1.62 | ± | 0.18 |  | -1.67 | ± | 0.34 |  | -1.47 | ± | 0.15 |  | -1.82 | ± | 0.26 |  | -1.81 | ± | 0.09 |  |
| mid inferior | -1.57 | ± | 0.21 | -1.42 | ± | 0.21 |  | -1.33 | ± | 0.39 |  | -1.29 | ± | 0.14 |  | -1.44 | ± | 0.25 |  | -1.45 | ± | 0.20 |  |
| mid inferolateral | -1.38 | ± | 0.18 | -1.23 | ± | 0.24 |  | -1.19 | ± | 0.39 |  | -1.12 | ± | 0.12 |  | -1.32 | ± | 0.24 |  | -1.25 | ± | 0.05 |  |
| mid anterolateral | -0.99 | ± | 0.10 | -0.85 | ± | 0.21 |  | -0.94 | ± | 0.23 |  | -0.88 | ± | 0.22 |  | -0.96 | ± | 0.18 |  | -0.88 | ± | 0.05 |  |
| apical anterior | -0.60 | ± | 0.11 | -0.62 | ± | 0.21 |  | -0.51 | ± | 0.18 |  | -0.40 | ± | 0.20 |  | -0.64 | ± | 0.15 |  | -0.64 | ± | 0.15 |  |
| apical septal | -0.84 | ± | 0.17 | -0.98 | ± | 0.21 |  | -0.84 | ± | 0.15 |  | -0.87 | ± | 0.14 |  | -1.12 | ± | 0.21 | * | -1.08 | ± | 0.14 |  |
| apical inferior | -0.96 | ± | 0.16 | -0.82 | ± | 0.26 |  | -0.75 | ± | 0.21 |  | -0.79 | ± | 0.27 |  | -0.93 | ± | 0.28 |  | -0.79 | ± | 0.18 |  |
| apical lateral | -0.72 | ± | 0.13 | -0.56 | ± | 0.24 |  | -0.47 | ± | 0.21 | * | -0.41 | ± | 0.31 |  | -0.56 | ± | 0.12 |  | -0.44 | ± | 0.12 | * |
| apex | -0.20 | ± | 0.15 | -0.20 | ± | 0.06 |  | -0.25 | ± | 0.09 |  | -0.30 | ± | 0.08 |  | -0.36 | ± | 0.13 |  | -0.27 | ± | 0.12 |  |
